# Automatic configuration of the Cassandra database using irace

**DOI:** 10.7717/peerj-cs.634

**Published:** 2021-08-05

**Authors:** Moisés Silva-Muñoz, Alberto Franzin, Hugues Bersini

**Affiliations:** IRIDIA-CoDE, Université Libre de Bruxelles (ULB), Brussels, Belgium

**Keywords:** Databases, Automatic configuration, Parameter tuning, Hyperparameter tuning, Cassandra

## Abstract

Database systems play a central role in modern data-centered applications. Their performance is thus a key factor in the efficiency of data processing pipelines. Modern database systems expose several parameters that users and database administrators can configure to tailor the database settings to the specific application considered. While this task has traditionally been performed manually, in the last years several methods have been proposed to automatically find the best parameter configuration for a database. Many of these methods, however, use statistical models that require high amounts of data and fail to represent all the factors that impact the performance of a database, or implement complex algorithmic solutions. In this work we study the potential of a simple model-free general-purpose configuration tool to automatically find the best parameter configuration of a database. We use the irace configurator to automatically find the best parameter configuration for the Cassandra NoSQL database using the YCBS benchmark under different scenarios. We establish a reliable experimental setup and obtain speedups of up to 30% over the default configuration in terms of throughput, and we provide an analysis of the configurations obtained.

## Introduction

With the continuously increasing deployment of data-centered applications, the performance of the data collection and processing pipeline is a key factor to consider when developing high performing applications. In particular, databases form a crucial part of this pipeline, as they operate on its slowest part, the storage. The performance of a database depends on several factors, including the hardware it operates on, the data stored, and its parameter configuration (Cao et al., 2018). Databases in fact expose several choices that impact their performance, so that users and practitioners can choose the most appropriate configuration for their scenario (Lu et al., 2019). Databases are usually provided with a default configuration suitable for many different cases (Debnath, Lilja & Mokbel, 2008). Having been adjusted by the developers over the years, the default configuration is robust and often offers high performance for a variety of applications and hardware infrastructures. In many practical applications, however, the structure or amount of data, or the workload, are known, or can be estimated in advance. It is therefore possible to improve the performance of the database on a specific scenario, by tailoring the most appropriate configuration based on the data and workload of each specific case (Cao et al., 2018; Zhang et al., 2019).

While historically the task of finding the best configuration has mostly been a manual task, researchers began to explore automatic methods since at least 2003, at first trying to explicitly model the relationship of database parameters (Kwan et al., 2003). Along with the rise of automatic configuration methods in optimization and machine learning, and in general with the adoption of artificial intelligence techniques also in databases, the last years have seen a surge in automatic methods proposed also for databases (Zhou et al., 2020). Several techniques have been used, from design of experiments to neural networks to search algorithms (Lu et al., 2019). Automatic configuration methods offer several advantages (Stützle & López-Ibáñez, 2013). First, they unburden users and practitioners of the tedious and time-consuming manual task of finding the best configuration for a certain scenario. Second, users can only evaluate a limited set of alternatives in manual experiments, injecting their own biases in the process and likely obtain very suboptimal results. Consequently, obtaining good results with a manual configuration process requires a high expertise on both the database and the specific scenario, while automatic configuration methods make this task accessible also to non-experts. Automatic methods, therefore, can be expected to find better configurations than manual trial-and-error experiments, with significantly less human effort (Stützle & López-Ibáñez, 2013).

The majority of the works proposed attempt to either evaluate configurations that are representative of the entire parameter space, or to learn some statistical model of the relationship between the parameter configuration of the database and performance obtained (Lu et al., 2019; Oh & Lee, 2005; Debnath, Lilja & Mokbel, 2008; Duan, Thummala & Babu, 2009; Rodd, Kulkarni & Yardi, 2013; Zheng, Ding & Hu, 2014; Mahgoub et al., 2017b). The application of these methods, however, requires great care. The evaluation of a database configuration is a heavy task: it requires a considerable amount of operations to be considered reliable, due to the stochastic nature of the task and to the hardware and software techniques to improve the performance, such as caching. Very short experiments may not be representative enough of the database performance (Zhu et al., 2017a). Therefore, configuring a database is an extremely computationally heavy task, that requires users to carefully balance the computational effort with the quality of the results, both in terms of efficiency and reliability of the final configuration obtained. Furthermore, attempts to model the relationship between parameters and the results cannot possibly include, or measure, all the factors that impact the performance of the database. For example, the specific hardware specifications, or the network characteristics in case of a distributed database, have an impact on the database performance, yet it is impossible to both model all such factors, and to maintain feasible computational requirements for the tuning task (Kuhlenkamp, Klems & Röss, 2014). In other words, we want to invest as little time and computing power as possible, to find a configuration that consistently gives the best results; and this unfortunately imposes a limit on the number of evaluations we can perform, which in turn prevents the observation of enough data to learn a good statistical model of the database performance. Attempts to devise a clever methodology that satisfies all these requirements can easily turn into complex algorithms, where several different techniques are combined. Last, a model of said relationships for a specific task or database is not transferable to, for example, a different database (Bao et al., 2018; Kuhlenkamp, Klems & Röss, 2014).

A certain number of works consider instead the database configuration problem as an optimization problem over a (potentially) mixed space of variables (Zhang et al., 2019; Li et al., 2019). While these methods are, in principle, database-independent, they are very complex. Developing an efficient optimization algorithm requires also to evaluate design choices and to fine tune its parameters, a task that effectively requires to repeatedly evaluate the performance of the algorithm in configuring a database, with an explosion in computational demands. Moreover, design choices and parameter values that are good for one database or scenario are not necessarily good for a different one.

We instead advocate an alternative, model-free approach that makes use of an easy-to-use, well-tested, general-purpose configurator to find the best settings for a database. Finding the best configuration for a database is, conceptually, no different than finding the best configuration for any other software or algorithm for which the quality of the output can be measured (Birattari, 2004; Bergstra et al., 2011). This problem arises in several other fields, and has been the subject of several lines of research, that produced powerful methods (Hutter, Hoos & Leyton-Brown, 2011; Bergstra & Bengio, 2012; Hutter et al., 2009). In particular, we use the irace R package to find the best configuration of the Cassandra database for given scenarios (Birattari et al., 2002; López-Ibáñez et al., 2016). irace is a state-of-the-art configurator, used to improve the performance for several applications, from optimization algorithms to machine learning, from compilers to the automatic design of algorithms. irace is as a model-free black-box stochastic optimizer, so it does not need to assume any statistical model of the relationship between the parameters and the target algorithm performance (the database, in our case), and it can be applied to any parameterized algorithm or system. It is also easy to use, since it requires only to set up few scripts and configuration files, without the need to implement complex algorithmic solutions. We show how irace can improve over the already high-quality default configuration, and that the potential for improvement increases the more specific the scenario is.

Our contribution is three-fold. We first separate the tasks of evaluating a configuration and configuring a database, and devise an experimental setup that is optimized for both speed and consistency. We perform systematic experiments to determine the best trade-off between consistency of the results and speed. We also show that experiments performed on our setup generalize well to heavier scenarios. We then show that irace is a solid method for automatically configuring a database with little manual intervention, obtaining significant performance improvements with respect to the default configuration with a relatively limited number of evaluations. Finally, we analyze the configurations obtained in the various cases, to give indications about how to perform future configuration tasks. Our experimental setup is provided as part of the Supplemental Material (Silva-Muñoz, Franzin & Bersini, 2020). Our approach is completely general, and can be applied to different relational and NoSQL databases with little effort. In this work we choose to study the performance of irace on the Cassandra database, one of the most popular NoSQL databases, used in several real-world applications such as Internet of Things, genomics, or electric consumption data (Cassandra, 2014; Duarte & Bernardino, 2016; Daz, Martn & Rubio, 2016; Mahgoub et al., 2017a; Le et al., 2014; Aniceto et al., 2015; Pinheiro et al., 2017). We measure the performance in terms of throughput using the YCSB benchmark (Cooper et al., 2010; Wang & Tang, 2012), observing a speedup of up to 30% over the default configuration.

In the next section we review existing automatic approaches to configure databases. In “Materials” we describe Cassandra and YCSB, together with the irace configurator and the general experimental setup. In “Experimental Results” we report our experiments to find a consistent experimental setup and to evaluate irace as configurator for databases. We then conclude outlining future research directions in “Conclusions”.

## Background and Literature Review

In this section we briefly review the parameter tuning problem and the methods proposed in the literature to automatically configure database systems, with their limitations. We can classify the existing works in two main categories, model-based and search-based methods.

### Parameter tuning

The task of parameter tuning, algorithm configuration, or (in machine learning) hyperparameter tuning consists in finding the configuration of a given software or algorithm that can obtain the best results for a given scenario (Birattari, 2004). Formally, for an algorithm }{}{{\rm {\cal A}}_{\rm {\cal P}}} for a certain problem }{}{\rm {\cal P}}, with *k* parameters \Theta = {\theta _1},{\theta _2}, \ldots ,{\theta _k}, a set of instances }{}{\rm {\cal I}} of }{}{\rm {\cal P}}, and a measure of the performance }{}m:\Theta \times {\rm {\cal I}} \mapsto {\rm {\open R}} to be (without loss of generality) maximized, the goal is to find }{}{\Theta ^*} = arg{\kern 1pt} maxm(\Theta ,{\rm {\cal I}}).

It is a stochastic black-box optimization problem that arises in many fields where the performance of a certain algorithm of software is crucial, and can be controlled by the user with the algorithm or software parameters. Databases are an example where this task is clearly relevant. They come with a set of parameters that impact their performance at various levels, such as the size of caches and buffers, or the number of concurrent operations allowed.

Parameter tuning is a hard problem in practice. It involves a mixed space of variables, as parameters can be of integer type, real-valued, categorical, representing unordered alternative choices, or ordinal, where a relationship between the parameter values can be established (for example, a parameter may take a value among {low, medium, high}). Parameters can also be dependent on each other, where for example parameters *p*_*a*_ is used only if another parameter *p*_*b*_ takes a certain value; and even when this is not the case, parameters interact in ways that are difficult to understand even for domain experts. A tuning is also strongly dependent on the scenario, such as the metric we use to evaluate the configurations, or the experimental setup.

We distinguish between *offline* parameter tuning, where the goal is to find one configuration of parameters to apply when first deploying the database, and *online* parameter tuning where instead the task is to modify the configuration at runtime, to adapt the database to a changed scenario (*e.g*. a different workload).

#### Scope of parameter tuning for databases

The use of artificial intelligence (AI) techniques has been increasingly replacing traditional methods based on human effort and heuristics in the deployment and management of database systems. In Zhou et al. (2020), AI techniques for databases have been classified in five broad areas, three of which are related to the optimization of the performance. These are database configuration, database optimization, and database design, listed in increasing order of complexity.

Database configuration refers to the fine-tuning of a certain set of operations to improve the performance. This includes the configuration of the database parameters (Zhu et al., 2017a; Van Aken et al., 2017; Zhang et al., 2019; Li et al., 2019), but also the index selection (Valentin et al., 2000; Schnaitter et al., 2007; Pedrozo, Nievola & Ribeiro, 2018) and view advisor (Zilio et al., 2004; Jindal et al., 2018; Yuan et al., 2020) that optimize the performance when accessing the data, and the query rewriting that manipulates the queries provided by the user in order to make them more efficient (Mahajan, Blakeney & Zong, 2019; Chavan et al., 2011; Baik, Jagadish & Li, 2019).

Database optimization involves several components of the database at the same time. It includes for example cardinality and cost estimation (Dutt et al., 2019; Marcus et al., 2019), that is, estimating how many rows will be accessed when executing a query, and how many resources will be used, join order selection (Stillger et al., 2001; Krishnan et al., 2018), to find the most efficient way of performing a set of join operations.

AI-based techniques for database design can instead be used to assist or replace human operators when designing a database, exploring a wider set of alternatives than would be possible in a manual task. This includes using machine learning algorithms to learn indexes and data structures (Kraska et al., 2018; Wu et al., 2019). Another important application is learning the patterns of transactions, in order to predict future operations and schedule them more efficiently (Ma et al., 2018; Sheng et al., 2019).

In this work we consider (offline) parameter tuning, one of the basic of database configuration. Before presenting our approach in detail, we first review the existing techniques proposed in the literature.

### Model-based methods

The first methods proposed for automatic database configurations tried to devise an explicit relationship between database parameters, or a subset of them, and the database performance. Kwan et al. (2003) explicitly provide a mathematical formulation of this relationship for DB2, and suggest an initial configuration. It is however unclear from their work if they consider all the parameters, or only a subset. Sullivan, Seltzer & Pfeffer (2004) devise an influence diagram involving 4 parameters of BerkeleyDB, and recommend a plausible interval for those parameters; (Dias et al., 2005) propose an analogous approach for the Oracle database. The main limitation of these works is the impossibility to include in the analysis all the possible factors that impact the performance, such as the hardware configuration, the data, or the workload.

Identifying the parameters that affect the performance the most, is a very important task for both obtaining an efficient database and being able to interpret the results. Several methods based on statistics and design of experiments have been proposed to this task. Oh & Lee (2005) use the Pearson correlation coefficient to determine the 4 parameters that impact the memory efficiency of Oracle the most. Debnath, Lilja & Mokbel (2008) use Plackett-Burman design to rank the parameters of PostgreSQL according to their importance. Babu et al. (2009) use adaptive sampling to tune two parameters related to the memory use of PostgreSQL. Duan, Thummala & Babu (2009) combine Latin Hypercube Sampling and Gaussian Processes to configure a database, and provide the first experimental results of a method on two different databases. In particular, they configure up to eleven parameters for PostgreSQL and MySQL. In 2017, (Van Aken et al., 2017) instead propose a model based on Gaussian Processes and gradient descent to configure the parameters of PostgreSQL, MySQL and ActianVector.

Parameters can also be altered while the database is in operation. Storm et al. (2006) use cost-benefit analysis and control theory to tune and adapt the buffer pool size of DB2. Tran et al. (2008) apply memory replacement policies based on an analytical model to configure 4 parameters that impact the buffer size of PostgreSQL. Fuzzy logic has been also used to configure in an online fashion the buffer parameters of Oracle (Zhang, Chen & Liu, 2012; Wei, Ding & Hu, 2014).

Following their successes on other fields, (deep) neural networks have been used also for database configuration. Rodd, Kulkarni & Yardi (2013) and Zheng, Ding & Hu (2014) both use neural networks to tune parameters related to the memory management in Oracle. Tan et al. (2019) propose an analogous approach for online buffer tuning of PolarDB-X. Mahgoub et al. (2017b) use deep learning to generate a surrogate model of the performance of the database, and combine it with a genetic algorithm to configure at runtime both Cassandra and ScyllaDB, identifying the most important parameters to which focus on. This work is then expanded to perform a more seamless online tuning (Mahgoub et al., 2019). Mahgoub et al. (2020) propose a similar approach, where this time a random forest is used to model the performance of a database in a virtualized environment, and combined with a genetic algorithm to jointly optimize the cloud virtual infrastructure and the database configuration. This solution is tested using Cassandra and Redis on Amazon EC2. Zhang et al. (2019) and Li et al. (2019) both apply deep reinforcement learning to configure MySQL, PostgreSQL and MongoDB.

### Search-based methods

While several model-based approaches have been proposed, not as many model-free works exist in the literature. Zhu et al. propose two dabatase-independent methods, the first one combining the divide-and-diverge sampling method with the bound-and-search algorithm, and the second one combining the Latin Hypercube Sampling method with the Random Search algorithm to configure the parameters of several databases, both relational and NoSQL, including a big data one (Zhu et al., 2017a, 2017b). These methods, however, are very complex and not easy to implement.

Dou, Chen & Zheng (2020) model the database configuration problem as a black-box problem that they solve using mREMBO (modified Random EMbedding Bayesian Optimization) through the generation of an embedded space of smallest dimension using a random embedding matrix. This work configures the full stack, namely the OS kernel, the JVM and a set of Elasticsearch parameters. The limitation of this approach is that it was designed specifically for one type of database and kernel. Furthermore, when configuring the full stack configuration space for a specific database, the operation of other systems that coexist in this space can be affected as well.

### Discussion

Improving the performance of a database by configuring its parameters is an approach that has received significant attention in the last years, similarly to analogous works in thriving fields such as machine learning. In fact, the majority of the works we have collected rely on some statistical modeling of the relationship between the parameter configuration and the performance of the database.

However, model-based methods have some significant limitations, that hamper their applicability on different scenarios (Lu et al., 2019). Simple methods can be used to configure only few parameters, while more complex approached require large amounts of data to be effective. Unfortunately, as we are going to see in “Finding a Reliable Experimental Setup”, a consistent experimental setup is quite computationally expensive, yet it is necessary to obtain reliable measurements. Modeling an explicit relationship between parameters and performance omits relevant factors such as the hardware configuration used; and including those factors is likely to make the model complex to implement, cumbersome to train and difficult to understand. Finally, the performance measured after training a model on a specific scenario cannot be expected to apply to different scenarios, such as different data, different workloads, or a different hardware configuration (Cao et al., 2018; Kuhlenkamp, Klems & Röss, 2014).

On the other hand, the search-based methods proposed do not depend on a specific database, but are also complex ad-hoc methodologies. However, the configuration of a database is conceptually no different than the configuration of any parameterized algorithm or software, a problem that has received significant attention in the last years (Birattari et al., 2002; Bergstra et al., 2011). In particular, a lot of interest followed the explosion of machine learning methods and the recent trend in automated machine learning, that includes (hyper-)parameter optimization among its tasks (Hutter, Hoos & Leyton-Brown, 2011; Bergstra & Bengio, 2012; Hutter et al., 2009; López-Ibáñez et al., 2016).

We therefore follow a third approach, to use an off-the-shelf, easy-to-use, general purpose tuner, namely the irace configurator, described in the next section.

## Materials

### The Cassandra database

Cassandra is a distributed NoSQL database designed to handle large amounts of unstructured data (Cassandra, 2014; Wang & Tang, 2012). Initially designed at Facebook by combining some characteristics of Google’s Bigtable and Amazon’s Dynamo,Cassandra has then become one of the most popular NoSQL databases.

Cassandra has a wide-column data store model, using columns as the basic data unit and structuring the data following the concept of column families.

One of the most important characteristics of Cassandra is its peer-to-peer distribution architecture where each node acts as both master and server, without a single point of failure. All the operations can therefore be performed in a decentralized manner, and the nodes periodically exchange information on their status. This architecture, coupled with a fault-tolerant writing process and the replication of the data, grants a high service availability.

Cassandra provides a SQL-like language called Cassandra Query Language (CQL). Using this language it is possible to create and update the schema of the database, and access the data.

We use Cassandra for two main reasons. First, Cassandra is one of the most used and best performing NoSQL databases today, with applications in several different domains (Duarte & Bernardino, 2016; Daz, Martn & Rubio, 2016; Mahgoub et al., 2017a; Le et al., 2014; Aniceto et al., 2015; Pinheiro et al., 2017). Second, the existing documentation is very complete, and it allows to easily replicate and generalize the experiments carried out in this work.

### The YCSB benchmark

The Yahoo! Cloud Serving Benchmark (YCSB) is one of the most popular benchmarks to assess the performance of relational and NoSQL database management systems (Cooper et al., 2010; Abubakar, Adeyi & Auta, 2014; Abramova, Bernardino & Furtado, 2014).

This tool consists of two parts: the record generator and the workload generator. The record generator allows to define the characteristics of the data stored in the database, specifying for example the length and type of the records and the distribution of the data when inserted. In this way it is possible to replicate a specific database scenario, by using the same kind and amount of data of real cases, without using the actual data.

The workload generator defines the quantity and type of operations to be executed. Like for the record generator, it is possible to replicate real-world cases by specifying the proportion of different operations from the following set: (i) read, which gets the value contained in a record, (ii) update, that overwrites the value contained in a record, (ii) scan, that reads all or a subset of the records in a table, (iv) insert, which adds a new record to the database, and (v) read-modify-write, which alters the value of a record by executing three successive operations as an atomic one. While the user can specify any workload, YCSB has a set of six default workloads.

- Workload A has a 50/50 read and update ratio.
- Workload B is a “read mostly” workload, with a read and update ratio of 95% and 5% respectively.
- Workload C is a read-only workload.
- Workload D is a “read latest” workload. This workload has 95% read and 5% insert operations, and the most recently inserted records are the most likely ones to be read.
- Workload E has 95% scan and 5% insert operations.
- Workload F is a read-modify-write workload. Half of the operations are read, while the other half consist in read-modify-write operations.

For different applications, it may be possible that some records are accessed more frequently than other ones. Thus, in YCSB it is possible to select the most probable distribution of the records that will be accessed by the operations. The three options are: (i) uniform, where each record has the same probability of being accessed; (ii) Zipfian, that increases the probability of accessing records selected in the past, and (iii) latest, that increases the probability of accessing the most recently inserted records. We use the default policy of YCSB, the Zipfian distribution, for the majority of the operations. The exceptions are Workload D, where a latest policy is used, the scan operations, that select a subset of the records in a table with a uniform distribution. In YCSB it is possible to run operations in parallel using multi-threading.

The use of YCSB has several advantages. First, it is possible to recreate various situations, by using alternative combinations of data and workload specifications. Second, using YCSB it is possible to easily generalize our approach to different databases. Finally, YCSB generates a comprehensive report, than can be used to analyze the status of the database.

### The irace configurator

We use the general purpose configurator irace, an implementation of the *iterated racing* algorithm freely available as an R package (López-Ibáñez et al., 2016; Birattari et al., 2002). Initially developed as a tool for optimization algorithms, it has been applied also to different domains, such as machine learning, automatic algorithm generation, or the configuration of the GCC compiler. In this work we investigate its efficiency on the task of configuring a database; the peculiar aspects of this task will be presented in “Finding a Reliable Experimental Setup”.

To perform a tuning, irace needs a *target algorithm*, its list of parameters with their type and possible values, and a set of *instances* to benchmark the configurations. In our case, the algorithm will be the Cassandra database, and the instances will be sets of database operations as defined by different YCSB workloads.

The basic routine of irace, called *race*, can be thought as a competition to select the best configuration among a set of candidate ones (Maron & Moore, 1997). The candidate configurations are evaluated on the same benchmarks, and a statistical test is used to eliminate from the race the ones performing poorly. The rationale for this process is that evaluations are expensive, and it is better to focus the limited budget of evaluation on the most promising candidates, rather than wasting it on poorly performing ones.

**Figure 1:**
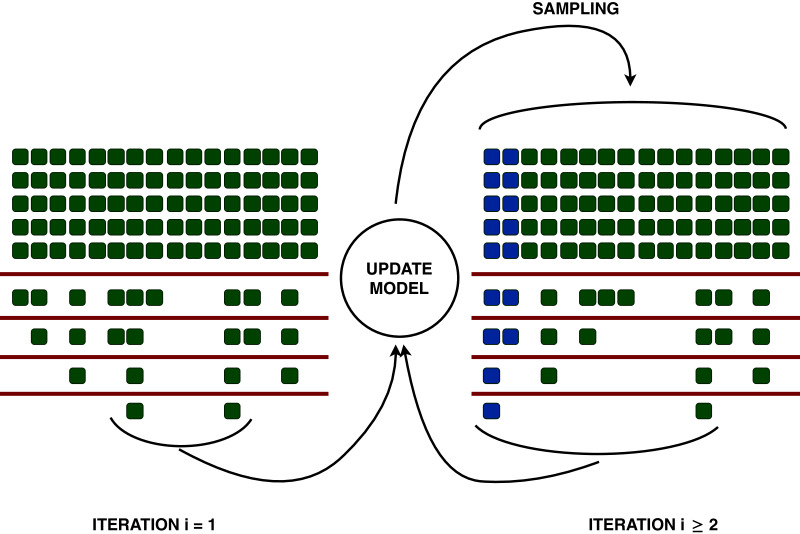
Representation of the racing process of irace. Each column is a different configuration, and each row is an instance evaluated. After a certain amount of instances, the algorithm discards the configurations that perform statistically worse than the best one, according to a Friedman analysis of ranks test (corresponding to the red lines). Instance after instance, the algorithm proceeds to benchmark surviving configurations, and discard the ones that perform poorly, until either the experiment budget for the race is exhausted, or a minimum number of surviving configurations remain. These surviving configurations, called elites, are used to update the model, to sample new candidate configurations around them. The elites (in blue) and the new configurations are evaluated in a second race. This process iterates until the total experimental budget of irace is consumed.

More precisely, irace starts with a set of configurations generated uniformly at random (possibly including some configurations provided by the user, such as a default configuration); each configuration is benchmarked on a sequence of instances (in our case, of workloads) until a certain amount of results have been collected for each configuration. At this point, a statistical test identifies the worse configurations, and removes them from the lot. The race continues repeating this process of evaluation and statistical elimination, until either the budget of evaluation for the race is exhausted, or a minimum number of configurations remain. The surviving configurations are then used as seeds to generate a new set of candidate configurations around them, to be benchmarked with a new race. This process iterates until the total amount of experiments is used. The process is represented in Fig. 1. This is a very efficient process, in that a configuration is evaluated on an instance only once, as in virtually any practical application the evaluation of the configurations takes almost the entire time. For a more detail description of irace, we refer to (López-Ibáñez et al., 2016).

We choose irace for several reasons. First, its efficiency has been demonstrated on several different scenarios. While developed primarily for the tune of optimization algorithms, irace has been successfully used also to configure machine learning algorithms, optimize code compilers, and automatically design new state-of-the-art optimization algorithms with several hundreds of parameters (Miranda, Silva & Prudêncio, 2014; Pérez Cáceres et al., 2017; Pagnozzi & Stützle, 2019). Second, its ease of use makes its use suitable also to non-experts. Being a black-box optimizer, irace does not require any insight about the inner working of our target algorithm. Therefore, we can seamlessly apply it to configure Cassandra, but also any other database we should need to tune. In this regard, irace has an advantage over model-based methods, as it does not need to assume statistical models about the impact of the parameter configuration, nor large amounts of data to train them. Conversely, at the moment it is difficult to make use of the data from past experiments in new scenarios.

### General experimental setup

We run our experiments in the Google Cloud infrastructure using n1-standard-8 machines, with eight virtual CPUs, 30 GB of memory and a 20GB persistent disk.

We use Cassandra version 3.6, that exposes several parameters that can impact the performance of the database, of categorical, integer, and real-valued type. The list of 23 parameters considered is reported in Table 1, with the parameter ranges. Using YCSB version 0.17, for each experiment we create a table with ten varchar fields of 100 bytes each.

**Table 1:** The 23 parameters that impact the performance of Cassandra. Parameters of type c are categorical, representing alternative choices; parameters of type i and r take, respectively, integer and real values. Parameters in boldface are the most important ones for the performance of Cassandra, as determined in Mahgoub et al. (2017b).

Parameter	Type	Values
**Concurrent writes**	i	[8, 64]
**File cache size**	i	[256, 2048]
**Memtable cleanup**	r	[0.1, 0.9]
**Concurrent compact**	i	[2, 16]
**Compaction strategy method**	c	(Leveled, SizeTiered)
Num tokens	i	[2, 256]
Concurrent reads	i	[8, 64]
Replication factor	i	[2, 11]
Memtable heap space	i	[1024, 3072]
Memtable allocation	c	(heap_buffers, offheap_buffers, offheap_objects)
Row cache size in mb	i	[0, 16]
Sstable open interval	i	[0, 100]
Trickle fsync	c	(True, False)
Inter dc stream	i	[100, 400]
Key cache size	i	[0, 200]
Stream throughput	i	[100, 400]
Row cache save	i	[0, 120]
Column index size	i	[32, 128]
Compaction throughput	i	[16, 64]
Memtable offheap space	i	[1024, 3072]
Commitlog segment	i	[16, 64]
Mem flush writers	i	[2, 16]
Index summary	i	[75, 150]

The measure we use to evaluate the configurations is the throughput, that is, the number of transaction per second: a good configuration yields a higher throughput than a poor one, so our goal is to find the configuration that maximizes the throughput.

The version of irace is 3.4, with budgets of 500, 1,000 and 2,000 experiments per tuning. Real-valued parameters use a precision of 2 decimal digits. An instance is a pair (YCSB workload, random seed). The statistical test used to discard poor-performing configurations is the Friedman test. The additional details on the setup are determined experimentally and discussed in “Finding a Reliable Experimental Setup”.

The basic setup of irace consists of three main components: (i) the parameter file, that contains the definition of the Cassandra parameters we configure; (ii) the scenario file, with the configuration of irace, including the tuning budget; and (iii) a script called target-runner that performs an atomic evaluation of Cassandra with a given configuration. The target-runner takes in input the candidate parameter configuration and the instance, evaluates it, and returns the throughput recorded. Optionally, a file containing user-defined configurations can also be provided: we use this file to include the default Cassandra configuration in some of our experiments. A full experimental setup to reproduce our experiments is included in the Supplemental Material (Silva-Muñoz, Franzin & Bersini, 2020).

Following the guidelines for evaluating a database, we can claim that our evaluation is fair (Raasveldt et al., 2018). We test the same database, under the same benchmark, on the same hardware. The significance of the evaluations is determined experimentally. Our goal is to evaluate the potential of a general purpose configurator to find configurations that improve over the default one, so each test is performed in the same way, and we report statistical comparisons of the results.

### List of experiments

Here we briefly introduce the list of experiments performed. In “Finding a Reliable Experimental Setup” we complete the experimental setup, by determining experimentally how much data and how many operations should be used, and what is the impact of some choices about how experiments are run on the results obtained. The goal is to obtain a setup that obtains consistent and scalable results, in the shortest possible time. Since a tuning consists in a sequence of evaluations performed under partially different conditions, the assurance about the meaningfulness of the results is even more important than the throughput obtained.

In “Automatic Configuration of Cassandra” we evaluate irace as a configurator for Cassandra. We consider different scenarios: (i) the six default YCSB workloads, (ii) YCSB Workload A, and (iii) YCSB Workload E. With the first one, we try to find a configuration that can improve over the default one for several cases at the same time. With the second and third scenario, we want to observe what is the impact of focusing on a specific case, respectively one for which Cassandra is well suited, and one where Cassandra struggles (Abramova, Bernardino & Furtado, 2014). Our set of scenarios is representative of different real world cases, where the user needs either flexibility over several possible scenarios, or, on the contrary, the user knows precisely the load of the database and needs a configuration tailored for the specific case. We test the potential of irace with increasing tuning budget, on the whole set of parameters and on a restricted one, and observe when and how it is convenient to exploit the high quality of the default configuration. Each configuration obtained is also applied to a heavier load, to test the validity of our experimental setup, and the general applicability of the tuning process.

Finally, we analyze the configurations obtained, and discuss them in relation with the default configuration and the scenario for which they were obtained.

## Experimental Results

### Finding a reliable experimental setup

Finding the best configuration of a database for a given scenario typically requires several experiments, each one requiring to benchmark the database with a different configuration for some time. These experiments are quite computationally expensive, and we usually cannot or do not want to run each of them at length. Unfortunately, short experiments cannot be considered reliable, as they are likely to give different results when repeated identically.

When configuring an optimization or machine learning algorithm, very often we can afford to run several experiments. In this case, however, each experiment takes a relatively long time, and it is more difficult to parallelize, so we need to use as few experiments as possible.

The first step in configuring a database is therefore to devise a minimal experimental setup that will allow users and practitioners to obtain consistent results. There are in fact several factors that impact the performance of a database. Some of these factors, such as the machine or the workload, are usually part of the problem definition. The parameter configuration of the database that yields the best performance for the given scenario is the factor we want to intervene on. There are however other possible spurious factors that impact our observation of the performance, caused by the intrinsic inner working of the database. In fact, our measurement of the throughput, or some other performance measure of choice, will also be affected by how much data we use, for how long we run our benchmark to measure the performance, or how we configure our machine.

Here we study these spurious factors and how to mitigate their impact, in order to devise an experimental setup that can obtain consistent results. A full factorial analysis of the possible experimental setups is clearly infeasible, so we break this task down into five basic questions.

#### How many operations to use?

The first factor we consider is the number of operations, that is, the minimum number of operations necessary to do a representative experiment. Here we test the default configuration of Cassandra with the YCSB workload A and a fixed amount of 100K rows of data in the database, varying the number of operations. Each evaluation is repeated ten times. We seek to find the minimum amount of data that gives results representative of higher amounts.

**Figure 2:**
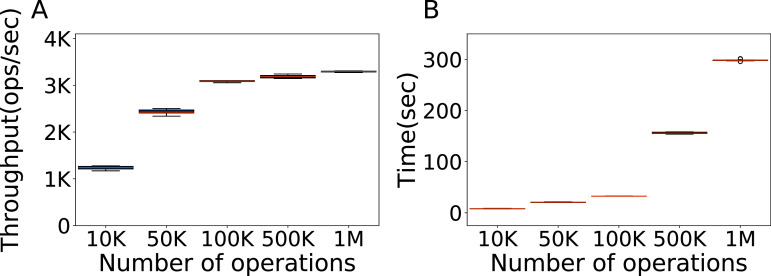
Number of operations to be used. Throughput obtained with 10K, 50K, 100K, 500K and 1M operations, with 100K rows of data, and the time each experiment takes. The results show the performance of Cassandra measured by testing the default configuration ten times on Workload A.

In Fig. 2 we show the throughput obtained with 10,000, 50,000, 100,000, 500,000 and one million operations, and the time it takes. Clearly, 100K operations are the best choice, obtaining a throughput that is both similar to the one of higher amounts of operations and of low variance, in a time similar to the time it takes to run 50K operations. Additional experiments with a one million rows of data, and with a number of operations ranging from 10K to 100K at intervals of 10K are reported in the Supplemental Material, and confirm that 100K is indeed a good value, for which we obtain results both consistent and representative of heavier loads, in a relatively short time (Silva-Muñoz, Franzin & Bersini, 2020).

#### How much data to use?

**Figure 3:**
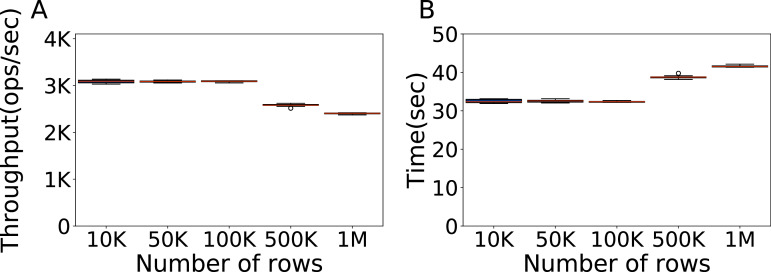
Size of the data to be used. Throughput obtained with 10K, 50K, 100K, 500K and 1M rows, with 100K operations per experiment, and the time each experiment takes. The results show the performance of Cassandra measured by testing the default configuration ten times on Workload A.

The next step is to determine the proper amount of data, in terms of rows in the database. We use again the Cassandra default configuration on Workload A and a fixed amount of 100K operations, varying the number of rows of data in the database. Each evaluation is repeated ten times. In Fig. 3 we show the throughput and relative time for 10,000, 50,000, 100,000, 500,000 and one million rows. The results indicate that using 100K rows of data we can obtain a good and consistent throughput, for the same time of lower amounts of data.

#### How many machines to use?

**Figure 4:**
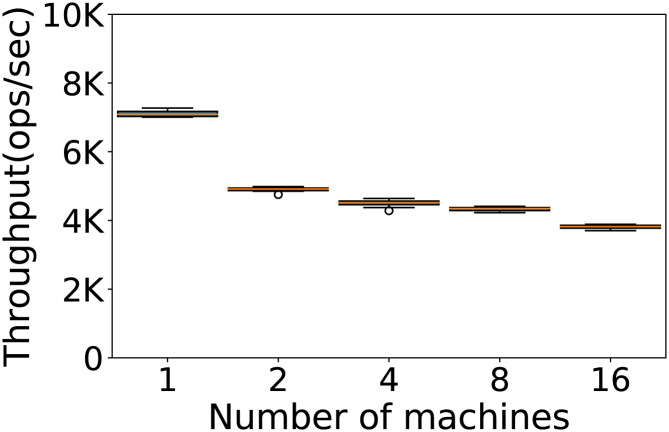
Number of machines to be used. Throughput obtained with 100K rows and 100K operations per experiment on 1, 2, 4, 8 and 16 machines. The results show the performance of Cassandra measured by testing the default configuration ten times on Workload A.

The number of machines we use in our experiments is another very important factor to consider to establish the framework where we run the experiments. We use the default configuration on Workload A, and 100K rows and operations. We evaluate the performance on 1, 2, 4, 8 and 16 machines. The throughput obtained is reported in Fig. 4.

The best results are obtained using a single machine. This is perhaps surprising, considering that Cassandra is a distributed database designed to linearly increase its performance along with the number of machines, but we note how our observations are consistent with what reported by other authors. (Haughian, Osman & Knottenbelt, 2016) report that Cassandra’s replication models degrade in both performance and consistency when more than one machine is used, in different cluster sizes and different workloads, due to Cassandra’s multi-master architecture. (Wang et al., 2014) also argue that a high replication factor in Cassandra can affect the performance. Finally, (Swaminathan & Elmasri, 2016) conclude that for databases of the moderate to large sizes the excessive distribution of data over different nodes and the overhead associated with the communication protocol can lead to a decrease in the performance of Cassandra. The results we obtained can also be in part explained by the use of a workload with a large portion of read operations, while a more write-heavy workload would probably benefit from a higher number of machines (Haughian, Osman & Knottenbelt, 2016).

On the other hand, we note that the absence of a single point of failure of a distributed architecture renders the database more robust, and may still be preferred in critical applications.

#### Is it better to reload the data base each time?

**Figure 5:**
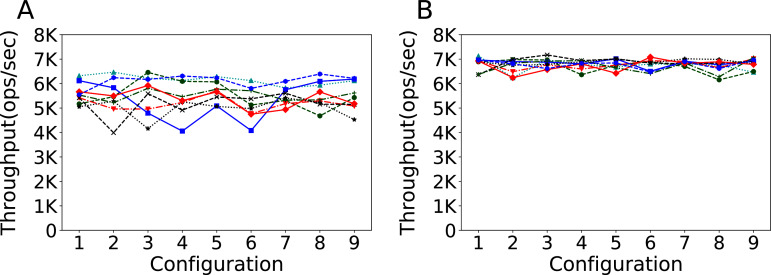
Warm and cold evaluations of different configurations. Throughput obtained with 100K rows and 100K operations per experiment. The results show the performance of Cassandra measured by running a series of nine different configurations nine times, changing the order of the configurations each run, without destroying the database after each experiment (left plot) and destroying and reloading the database for each run (right plot).

Another factor that can influence run performance is how data is loaded between runs. With these experiments we want to observe what effect it has on the configuration task, where several different configurations have to be evaluated sequentially. In Fig. 5 we observe the results obtained by nine different configurations, chosen according to a design of experiment strategy to cover the parameter space as much as possible, on the same experimental setup, when ran in different order without destroying the database between evaluations (warm evaluations, subfigure A) and destroying and reloading the database each time (cold evaluations that include the comparatively slow startup phase (Raasveldt et al., 2018), subfigure B). We obtain both a higher throughput and more consistent results when using cold evaluations.

In this case, the destruction and reloading of the database is not merely a feature of the experimental setup that affects the performance, but the only choice to not invalidate the whole tuning process. In fact, the infeasibility of the modification of the configuration in between runs would essentially devoid of meaning the throughput measured with warm evaluations.

This reliability comes, however, at the cost of the time needed for each evaluation. Warm evaluations on this setup take on average 50.2 s to complete, while cold ones take on average 64.5 s, *circa* 28% more. Overall, however, the evaluation time remains the 77% of the total time of each experiment, and this can be considered a reasonable price to pay for, in exchange, the assurance of a valid and consistent tuning process. We also note how, by virtue of their higher reliability, cold experiments obtain a higher throughput than warm ones.

#### Does the number of users impact the results?

**Figure 6:**
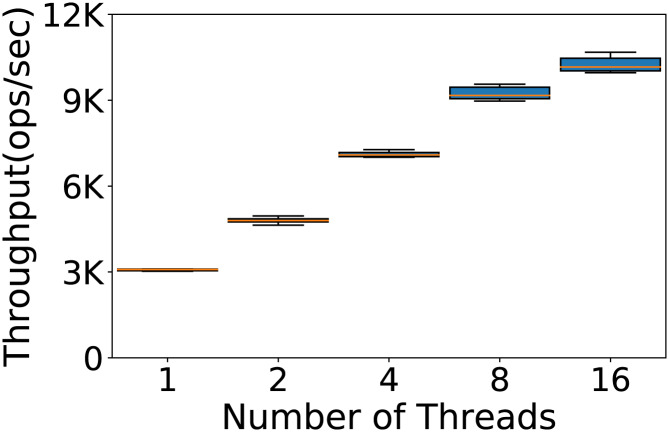
Number of YCSB threads to be used. Throughput obtained with 100K rows and 100K operations per experiment running the experiments with 1, 2, 4, 8 and 16 threads. The results show the performance of Cassandra measured by testing the default configuration ten times on Workload A.

YCSB can simulate the use of the database from different users by executing the operations in a multithreaded fashion. So far we used one thread to run our experiments, but we want to test whether the performance of Cassandra can depend also on the number of concurrent threads used. In Fig. 6 we report the throughput obtained testing 1, 2, 4, 8 and 16 YCSB threads with the default Cassandra configuration on Workload A, on a database with 100K rows and 100K operations. We see that Cassandra responds very well to the different number of threads. We choose to use 4 threads because of the very low variance in the results.

#### Conclusions

While the proportion of read and write operations is defined by the workload, their amount will determine the length of each experiment. Unfortunately, the impact of an inadequately low amount of operations will be overly affected by the initial loading of the database, something that can instead be considered negligible in a production setting. Likewise, for too little data the effect of caching will give unreliable observations.

The final experimental setup that we will use in the following Section is therefore composed of 100K rows and operations, for a database on a single machine. We use 4 threads in YCSB to simulate four users querying concurrently the database. Finally, the destruction and reloading of the database before every configuration evaluation will ensure a correct tuning process.

Based on our experiments, this setup is representative of heavier loads and is likely to scale well, while requiring a computational effort that is not much greater than lighter loads.

### Automatic configuration of Cassandra

Having obtained a reliable experimental setup, we can proceed with the automatic configuration of Cassandra using irace. For a tuning to be effective in a production environment, the training phase needs to resemble as much as possible the conditions of the production. We therefore consider three different cases, represented by three different workload conditions.

**W6** All the six YCSB workloads. With this experiment we aim to observe the capability of irace to obtain a configuration that outperforms the default one on a variety of applications.

**WA** YCSB Workload A. This is a workload with only read and update operations, where the default configuration of Cassandra performs particularly well.

**WE** YCSB Workload E. This is a scan-heavy workload, a case where the default Cassandra configuration does not excel.

We evaluate irace on every workload condition by configuring two sets of parameters: the set of 23 Cassandra parameters that impact the performance, reported in Table 1, and the five most important parameters, as determined in Mahgoub et al. (2017b) (in boldface in the table). In total we have therefore six tuning scenarios. We note that, using 23 parameters, the number of possible configurations is roughly 1.18 × 10^38^. For every scenario we perform three tuning tasks with increasing tuning budget, 500, 1,000 and 2,000 experiments, including the default Cassandra configuration in the initial set of irace candidate configurations, and three tunings with the same budget without including the default configuration. The tuning process with irace is described in “Materials”; the material to reproduce the experiments is reported in the Supplemental Material (Silva-Muñoz, Franzin & Bersini, 2020).

The final configurations obtained are tested on the same workload of the relative tuning, under the same experimental setup (100K rows in the database and operations, testing scenario TS1) and on a heavier setup (one million rows and operations, testing scenario TS2). Each final configuration is tested ten times. The results are reported in the following, in terms of speedup obtained with respect to the default Cassandra configuration under the same testing conditions. A higher boxplot thus means better results, while a boxplot whose average is below 0% indicates that the relative configuration performs worse than the default one.

In the Supplemental Material we include additional tables with the p-values resulting from pairwise Wilcoxon tests, for statistical significance, and additional plots comparing the configurations obtained by the tunings in the various scenarios with the default configuration (Silva-Muñoz, Franzin & Bersini, 2020).

#### Workload W6

**Five parameters**

**Figure 7:**
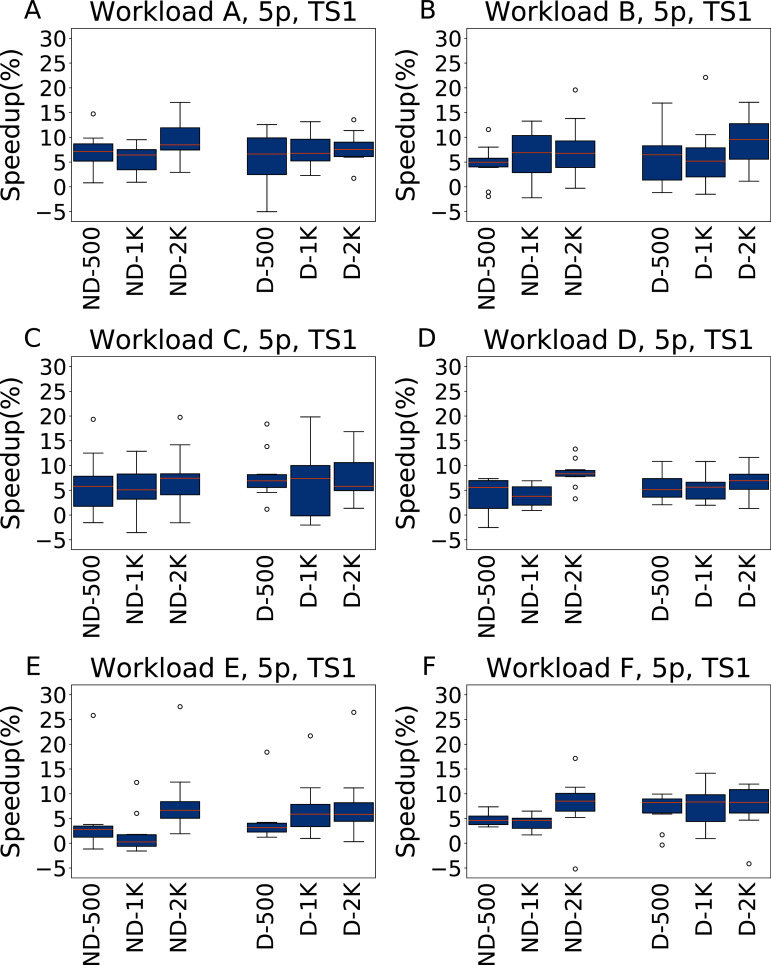
Speedup with respect to the default configuration obtained when tuning five parameters for all the six YCSB workloads (test scenario 1). The results show the measurement by testing the final configuration over each workload, repeating each experiment ten times. The boxplots report the speedup obtained by, from left to right, irace without the default configuration (ND-x, for budgets of 500, 1,000 and 2,000 experiments), and irace with the default configuration (D-x, for budgets of 500, 1,000 and 2,000 experiments).

The results for TS1 are reported in Fig. 7, divided by workload. Each plot includes the results obtained with and without the default configuration (boxplots D-*x* and ND-*x*, respectively, where *x* is the tuning budget used) using the three different tuning budgets.

In most cases irace finds a configuration that performs, on average, 5.9% better than the default Cassandra one. In general, there is not much difference between the tuning budgets 500 and 1,000, but a budget of 2,000 experiments can find configurations that are both better performing and that are more consistent in their results.

**Figure 8:**
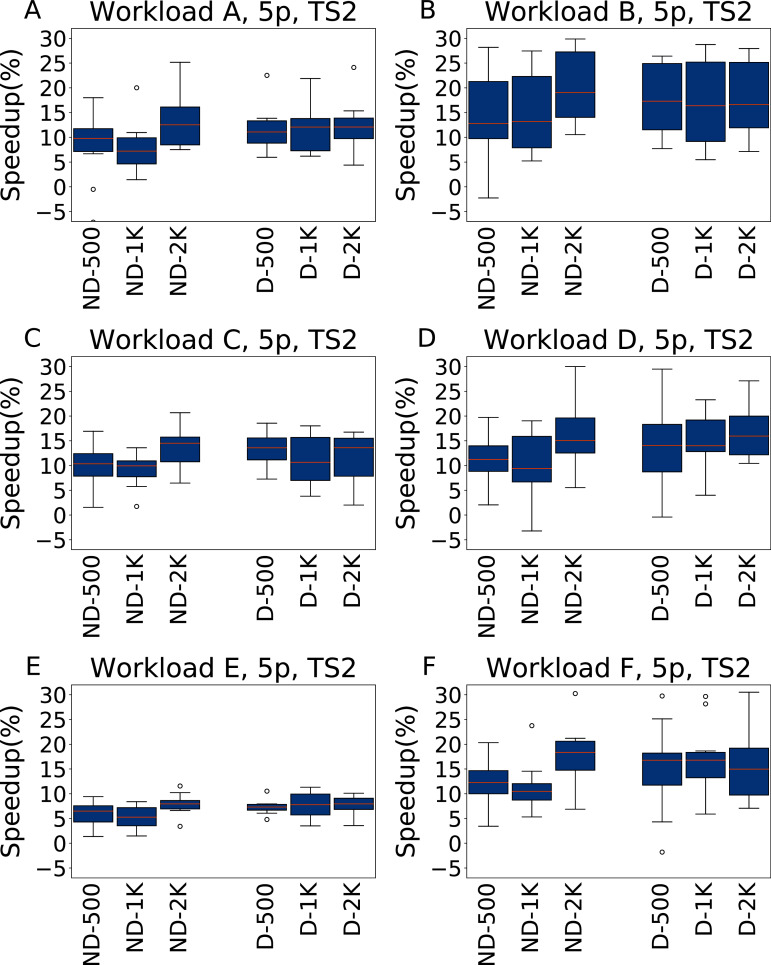
Speedup with respect to the default configuration obtained tuning five parameters for all the six YCSB workloads (test scenario 2). The results show the measurement by testing the final configuration over each workload, repeating each experiment ten times. The boxplots report the speedup obtained by, from left to right, irace without the default configuration (budgets of 500, 1,000 and 2,000 experiments), and irace with the default configuration (budgets of 500, 1,000 and 2,000 experiments).

The throughput improves in the heavier testing scenario TS2, reported in Fig. 8, where the average speedup is around 13%, albeit with higher variability. The main exception is observed for Workload E, the most dissimilar from the other workloads, where the configurations found by irace remain below 10% of speedup. In this case, however, there is a lower variance, and the speedup is consistently positive.

**Twenty-three parameters**

**Figure 9:**
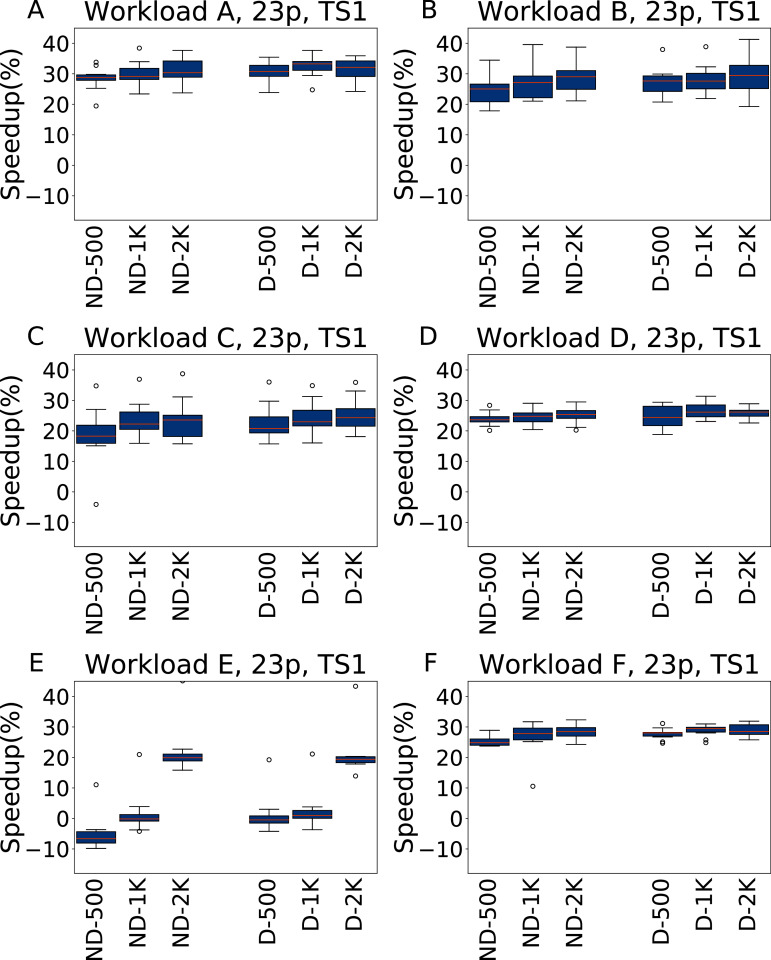
Speedup with respect to the default configuration obtained tuning 23 parameters for all the six YCSB workloads (test scenario 1). The results show the measurement by testing the final configuration over each workload, repeating each experiment ten times. The boxplots report the speedup obtained by, from left to right, irace without the default configuration (budgets of 500, 1,000 and 2,000 experiments), and irace with the default configuration (budgets of 500, 1,000 and 2,000 experiments).

**Figure 10:**
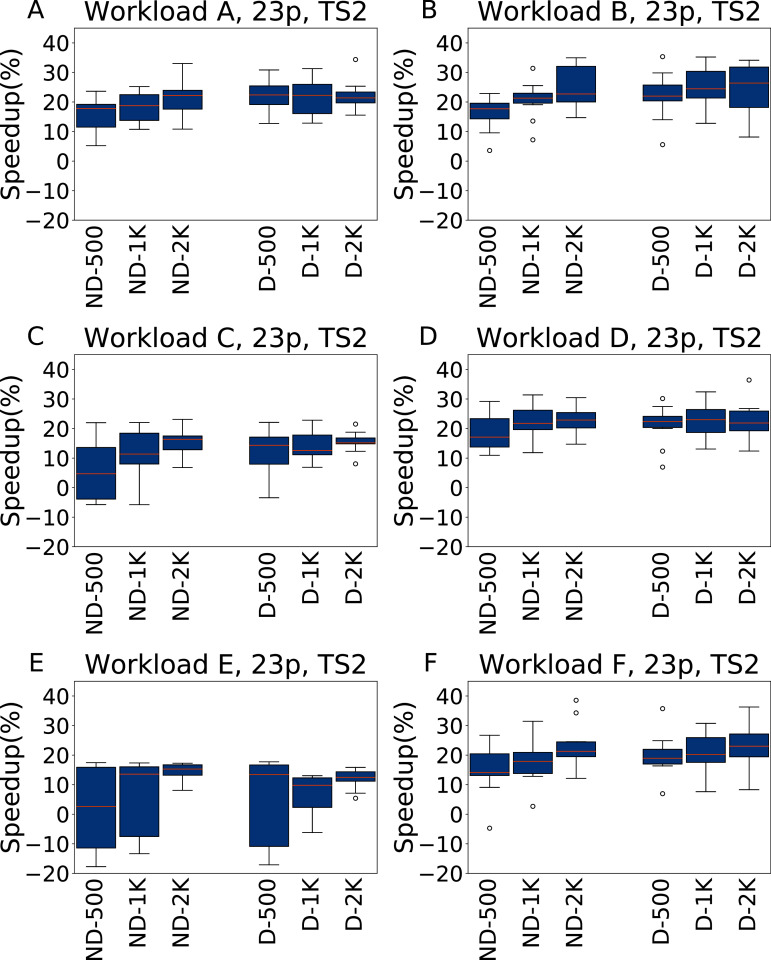
Speedup with respect to the default configuration obtained tuning 23 parameters for all the six YCSB workloads (test scenario 2). The results show the measurement by testing the final configuration over each workload, repeating each experiment ten times. The boxplots report the speedup obtained by, from left to right, irace without the default configuration (budgets of 500, 1,000 and 2,000 experiments), and irace with the default configuration (budgets of 500, 1,000 and 2,000 experiments).

In Figs. 9 and 10 we report the results for TS1 and TS2 respectively obtained across the 6 YCSB workloads when tuning the 23 Cassandra parameters. For TS1, on five workloads the average speedup is always between 20% and 30% over the default configuration, even for a tuning budget of 500 experiments. The only exception to this is Workload E, where for the lower budgets the speedup is negligible, or even absent (in the ND-500 case); However, even for this workload the configuration found with 2,000 experiments of budget obtain consistently a speedup or around 20%.

For TS2, instead, the speedup is lower, and less consistent, than for TS1, but still higher than when tuning five parameters. We explain this with the fact that while configuring 23 parameters allows to obtain, in general, better results than tuning only five parameters, this higher potential comes with the risk of overfitting. In particular, in this case the configurations obtained perform better when tested on the same amount of data and operations considered in the tuning.

**Analysis**

A statistical analysis, performed with a pairwise Wilcoxon test and reported in the Supplemental Material, confirms that in most cases, and for all the tunings with 2,000 experiments, there is a significant improvement over the default configuration. Only for Workload E and ND-500, the one most unfavourable for irace, the configuration found by the default configuration is significantly better, and in few tests there is a statistical equivalence between the results obtained.

Nonetheless, we obtained a very diverse set of configuration, and there is no value, for each parameter, that can be considered “the best” in itself. When tuning 23 parameters we observe the same effect: the value for the most important parameters in the extended set of parameters differs, sometimes significantly, than when tuning five parameters. This happens because of the interplay of the parameters, that is more important than the specific value of several of the parameters.

#### Workload WA

**Five parameters**

**Figure 11:**
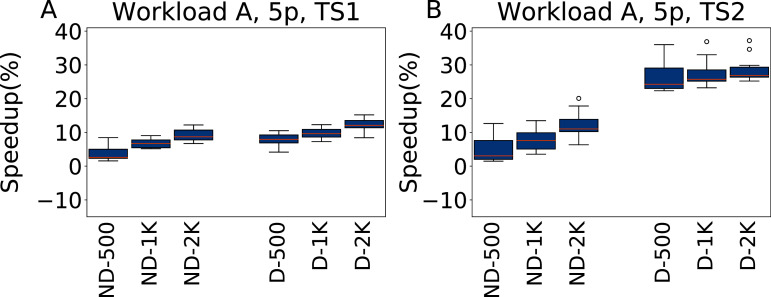
Speedup with respect to the default configuration obtained tuning five parameters for Workload A in test scenario 1 and test scenario 2. The results show the measurement by testing the final configuration over Workload A, repeating each experiment ten times. The boxplots report the speedup obtained by, from left to right, irace without the default configuration (budgets of 500, 1,000 and 2,000 experiments), and irace with the default configuration (budgets of 500, 1,000 and 2,000 experiments).

The results for tuning five parameters over Workload A are reported in Fig. 11, for both TS1 and TS2. Tuning for a single workload proves to be an easier scenario. irace improves over the default configuration even for a budget of 500 experiments, and higher budgets result in higher improvements. As Workload A is one where Cassandra performs particularly well with its default configuration, including this one in the tuning allows to obtain immediately very good results. The heavier testing scenario TS2 emphasizes these observations, with speedups up to 28.7% over the default configuration, whereas on TS1 we obtain at most an average of 12% improvement in the throughput.

**Twenty-three parameters**

**Figure 12:**
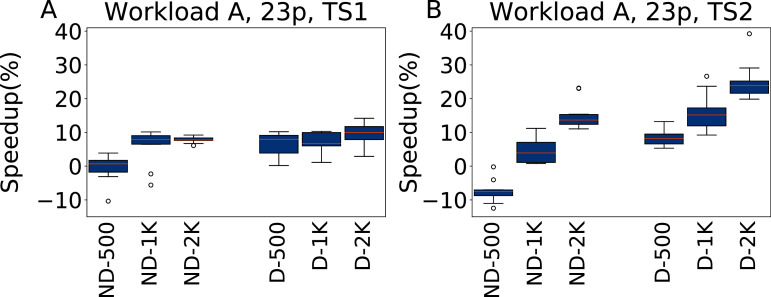
Speedup with respect to the default configuration obtained tuning 23 parameters for Workload A in test scenario 1 and test scenario 2. The results show the measurement by testing the final configuration over Workload A, repeating each experiment ten times. The boxplots report the speedup obtained by, from left to right, irace without the default configuration (budgets of 500, 1,000 and 2,000 experiments), and irace with the default configuration (budgets of 500, 1,000 and 2,000 experiments).

The results obtained tuning 23 parameters on Workload A are reported in Fig. 12 for TS1 and TS2. In this case the results are slightly less good than when tuning five parameters. The most noticeable difference happens for ND-500, that is even on average almost equivalent (for TS1) and worse (for TS2) than the default configuration. In this case, the much larger parameter space than on the five parameter case makes it more difficult to obtain a good configuration. The inclusion of the default configuration makes it instead possible to immediately converge the search to a good area of the parameter space, and to obtain consistently good results.

**Analysis**

In all the cases, a pairwise Wilcoxon test confirms that the results are statistically significant, except for ND-500 with 23 parameters, where the results are perfectly equivalent. For both 5 and 23 parameters the configurations are more uniform than in the W6 case, and more similar to the default configuration. This reflects the suitability of the default Cassandra configuration for scenarios with a balanced amount of read and write operations.

#### Workload WE

**Five parameters**

**Figure 13:**
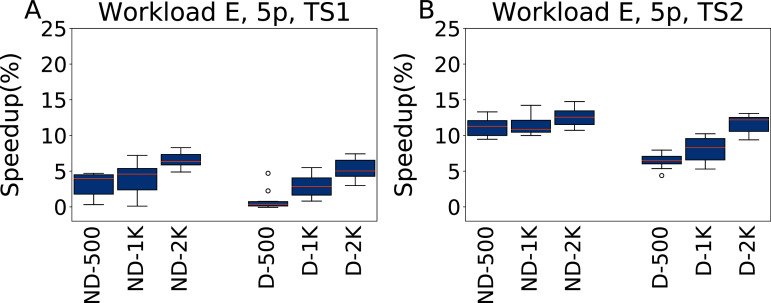
Speedup with respect to the default configuration obtained tuning five parameters for Workload E in test scenario 1 and test scenario 2. The results show the measurement by testing the final configuration over Workload E, repeating each experiment ten times. The boxplots report the speedup obtained by, from left to right, irace without the default configuration (budgets of 500, 1,000 and 2,000 experiments), and irace with the default configuration (budgets of 500, 1,000 and 2,000 experiments).

In Fig. 13 we report the results of the configurations obtained by irace when tuning five parameters under Workload E, evaluated on TS1 and TS2. Worklaod E is one where Cassandra does not excel, and this is reflected by the fact that the configurations ND-*x* perform better than the D-*x* ones for the same tuning budget. irace finds configurations that obtain speedups of up to 6.6% for TS1, and 12.5% for TS2. When including the default configuration in the tuning setup, it is still possible to obtain configurations as good as in the ND-*x* case, but it takes a higher budget for the search to steer away from the area of relative poor quality around the default configuration.

**Twenty-three parameters**

**Figure 14:**
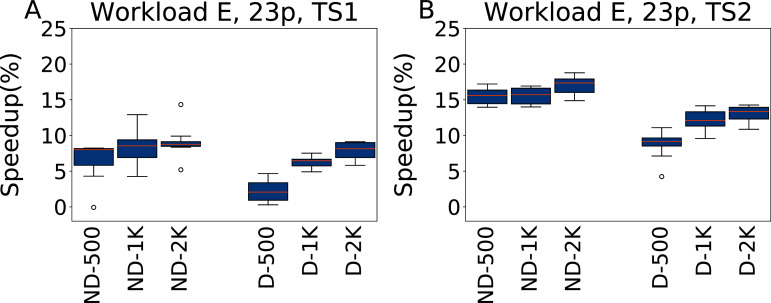
Speedup with respect to the default configuration obtained tuning 23 parameters for Workload E in test scenario 1 and test scenario 2. The results show the measurement by testing the final configuration over Workload E, repeating each experiment ten times. The boxplots report the speedup obtained by, from left to right, irace without the default configuration (budgets of 500, 1,000 and 2,000 experiments), and irace with the default configuration (budgets of 500, 1,000 and 2,000 experiments).

In Fig. 14 we report the speedups of the configurations found by irace tuning 23 parameters under Workload E, tested on TS1 and TS2. In this case the inclusion of all the parameters does not result in overfitting, but makes instead possible to obtain better results than when considering only five parameters, because finding a configuration that outperforms the default one in this case is easier than for other workloads. We obtain speedups of 9.1% for ND-2000 for TS1, and 17% for the same configuration for TS2. It is to be noted that ND-500 reports a speedup of 6.5% for TS1, which increases to 15.5% on the heavier testing scenario TS2. As when tuning five parameters, a larger budget allows configurations D-*x* to still obtain good results for higher budgets, even if they do not scale as much as the ND-*x* on TS2, reaching at most a speedup of 13%.

**Analysis**

Also in this case the results are always statistically significant, including for the configurations D-500 which are the ones that obtain the minimal improvement among the ones in this set of experiments. Like for Workload A, there is some uniformity in the configurations obtained; this time, the configurations are less similar to the default configuration than in the WA scenario, but not as much on the most important parameters as on the other parameters.

### Comparison with the Rafiki configurator

As part of the experimental results we include a comparison of the results obtained by irace with those of an state-of-the-art database configurator, Rafiki (Mahgoub et al., 2017b). Rafiki is a model-based methodology that builds a performance prediction model by training a deep neural network (DNN) from data collected in a set of preliminary experiments considering different configurations and workloads. A genetic algorithm is then used to find the best configuration for a given workload, using the DNN as a surrogate model to estimate the performance of the configurations generated.

For a fair comparison, we evaluate Rafiki under the same conditions we used in our experiments for irace. We consider therefore a budget for its initial set of experiments of 500, 1,000 and 2,000, using the same experimental setup obtained in “Finding a Reliable Experimental Setup”. We used the source code made available by the authors, and considered the five parameters setup (the ones selected as most important in Mahgoub et al. (2017b), and hardcoded in their implementation). Rafiki handles the workloads in the testing process differently from irace, so for an equal benchmarking we consider experiments on YCSB workloads A and E. The configurations obtained by Rafiki are then tested ten times for statistical evaluation.

**Figure 15:**
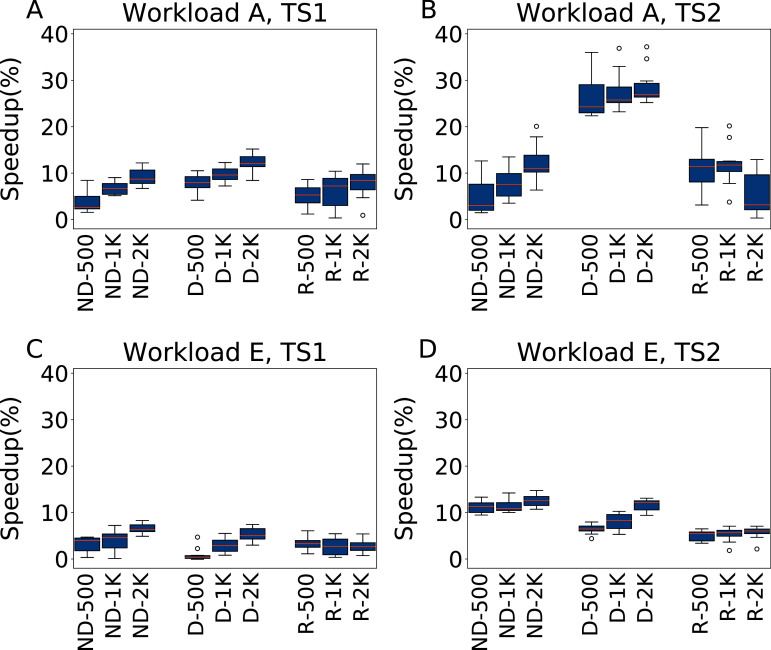
Comparison of speedups obtained by irace and Rafiki. Results in terms of speedup over the default configuration obtained by irace and Rafiki, using five parameters for Workload A (top row) and Workload E (bottom row), repeating each experiment ten times. Each boxplots reports the speedup obtained by, from left to right, irace without the default configuration (budgets of 500, 1,000 and 2,000 experiments), irace with the default configuration (budgets of 500, 1,000 and 2,000 experiments), and Rafiki (budgets of 500, 1,000 and 2,000 experiments).

The results are reported as R-*x* in Fig. 15 for workloads A and E, compared against the corresponding results obtained using irace including (D-*x*) and not including (ND-*x*) the default configuration as an initial configuration. The results of a pairwise Wilcoxon test analyzing the statistical significance of the different performance obtained with the two methods are reported in Table 2.

**Table 2:** Pairwise Wilcoxon test comparing the results in terms of throughput obtained using irace with those obtained using Rafiki, for five parameters. The first column of the table shows the workload (A or E from YCSB) and the test scenario in which the experiments were run (TS1 or TS2). The remaining columns show the statistical test results for budgets of 500, 1,000 and 2,000 experiments per tuning including (D-x) and not including (ND-x) the default configuration as an initial configuration for irace. Results in boldface indicate statistically significant results for a *p*-value threshold of 0.05.

Configuration	ND-500	ND-1000	ND-2000	D-500	D-1000	D-2000
WLA-TS1	0.193359375	0.556640625	0.431640625	**0.005859375**	**0.001953125**	**0.00390625**
WLA-TS2	**0.001953125**	**0.00390625**	**0.00390625**	**0.001953125**	**0.001953125**	**0.001953125**
WLE-TS1	0.845703125	0.556640625	**0.001953125**	**0.001953125**	0.76953125	**0.005859375**
WLE-TS2	**0.001953125**	**0.001953125**	**0.001953125**	**0.013671875**	**0.001953125**	**0.001953125**

For Workload A, TS1 we can see that the results are similar in terms of performance, where the relationship between budget and performance is established in all cases, with irace including the default configuration being the one that obtains slightly better results. The results obtained by Rafiki in this case are statistically equivalent to those obtained by irace without the default configuration. Under TS2, the D-*x* irace configurations show an evident improvement, with average speedups of 26.7%, 27.7% and 28.7% for the 500, 1,000 and 2,000 budgets respectively, while the results of the Rafiki show improvements of 11.4%, 11.9% and 5.4% for the same budgets. For Workload E, TS1 the results are of similar quality, with irace being statistically better for ND-2000, D-1000 and D-2000. Under TS2 the best option is irace ND-*x*, that obtains speedups of 11.2%, 11.4% and 12.5% for the 500, 1,000 and 2,000 budgets respectively, while the results of Rafiki show speedups of 5%, 5.3% and 5.6% for the same budgets.

The results can be explained by considering that irace is an offline configurator, whose goal is to learn one configuration, while Rafiki was designed to perform workload-dependent online tuning. Thus, in the tuning phase irace progressively intensifies the search around promising candidate configurations, while the experiment pool of Rafiki is entirely used to cover as much as possible the search space. The need for an observed workload in the tuning phase makes it also not possible to directly replicate our experiments of “Workload W6” with Rafiki without modifying its source code. For the same reason we could also not perform experiments with the whole set of 23 parameters.

## Discussion

The experiments presented in this section show how irace is effective in finding parameter configurations for Cassandra that outperform the default one for single and multiple different workloads. As expected, a higher budget finds better configurations. In many cases, a budget of 500 experiments is sufficient to improve over the default configuration. This does not always happen, in particular when tuning the whole set of 23 parameters relying on the default configuration in scenarios where it is not well suited, or, on the contrary, not relying on it in cases where it is well suited. On the other hand, with a budget of 1000 experiments we always found improving configurations.

While the computational cost of each evaluations is relatively high, this is, however, a remarkably low amount of evaluations, for a set of few dozens of configurations evaluated. Very similar configurations will obtain very similar results, and in this case the noise introduced by the stochasticity will probably have a higher impact on the throughput (which reinforces the need of a proper experimental setup). There is however evidence that the parameter landscapes are somehow “easy” to traverse for automatic configurators (Pushak & Hoos, 2018), while being clearly impossible to understand for human operators.

The time taken for each tuning on the machine described in “General Experimental Setup” is reported in the Supplemental Material. By doubling the tuning budget, the total tuning time roughly doubles too.

Whether including or excluding the default configuration from the tuning setup is beneficial for the final results depends on the workload considered. Cassandra, and its default configuration, obtain the best results for workloads with a mix of read and write operations: in these cases, the default configuration provides a good starting point that can be improved. In cases (such as Workload E) where Cassandra has lower performance, the default configuration slows down the tuning process.

Similar mixed indications come from the comparison between the results obtained when tuning 5 and 23 parameters. In many cases the inclusion of all the parameters makes it possible to fine-tune the database in a way that is not possible by using only five parameters, even the ones with the highest impact; this is a consequence of the interaction of the parameters, as observed also by authors of previous works (Duan, Thummala & Babu, 2009, Van Aken et al., 2017; Lu et al., 2019).

It is however possible that, in certain scenarios, the reduced search space generated by only five parameters makes it easier to find a good configuration, especially considering a limited tuning budget.

Therefore, while configurators, and generic-purpose ones such as irace in particular, make the configuration process accessible also for non-experts, a certain degree of domain expertise remains necessary to exploit the full potential of the tuning process, in particular when a limited tuning budget is used. By having reliable estimates of the data and workload, users can tailor the tuning for their specific case, in order to obtain the best results.

### Analysis of the default configuration of Cassandra

The default configuration of Cassandra is clearly a high-quality one, that has been carefully fine-tuned over the years by the developers to perform well on several different scenarios and hardware configurations. Nonetheless, as we have seen it is possible to consistently improve over it.

The configurations obtained for the workload scenario W6 are very different from each other, yet they consistently outperform the default one, sometimes by a high margin. It is clearly impossible even for an expert database administrator to manually craft such configurations. For example, the parameter memtable_cleanup_threshold takes a default value of 1/(*memtable*_*flush*_*writers* + 1), but in our final configurations we do not observe such relationship.

On more focused benchmarks, in particular WA, we can instead observe more consistent configurations, because of the narrower scope of the tuning, and some parameter values are more similar to the ones in the default configuration. For example, on WA the parameters concurrent_writes and file_cache_size_in_mb always take values close to the default setting of 32 and 512, respectively. On the other hand, memtable_cleanup_threshold and concurrent_compactors take very different values from the default configuration. These two parameters have an effect on the performance of read and write operations, and the different values are probably caused by the specific data format and workloads used in our tests. A higher value of memtable_cleanup_threshold translates in larger and less frequent flushes. The different value for concurrent_compactors reflects the fact that we have a lot of disk space in our machines; this cannot be assumed for a generic Cassandra installation, and this is the likely reason for the default conservative value of 2. Another factor that possibly contributed to those final configurations is the higher proportion of read operations over writes in Workload A, that makes the efficiency of reading operations more important than writing operations.

On WE the concurrent_writes and file_cache_size_in_mb parameters take consistent values across all configurations. We note how the high range of values now taken by parameters memtable_cleanup_threshold and concurrent_compactors reflects their reduced importance in this scenario, where there are no write operations. Similarly, when not starting the tuning from the default configuration the value of the compaction method in this case is Leveled, which is a better choice for reading operations, while starting from the default configuration, the value remains SizeTiered.

## Conclusions

In modern data-centric applications, the performance of the database is paramount, since a poor-performing database will affect the entire data processing pipeline. Finding the best parameter configuration for the database in use is therefore a very important task. Benchmarking a database configuration, however, is not a trivial task, since a balance between computational effort and significance of the experiments has to be found.

Configuring a database is far from a trivial task, due to the high computational cost of each experiment, the stochasticity of the results, the dependency from the hardware configuration and the benchmarking environment, and the intricacies arising from the internal workings of the database. While several methods have been proposed to this task, they require high amounts of data, are inadequate to model all the interactions between the factors that affect the performance of a database, or implement very complex algorithms.

In this work we have instead studied the potential of a general purpose automatic configurator, irace, to find high quality configurations for databases, and have reported experiments on different scenarios using the popular Cassandra NoSQL database. As a preliminary phase, we have devised an experimental setup that allows to obtain consistent results and is representative of heavier loads, at the minimum possible cost. We have observed how, while the performance that can be obtained obviously depends on the amounts of experiments performed, irace can find good quality configurations even with a limited tuning budget, in a huge configuration space. Thanks to the experimental setup we used, the configurations found can also scale very well to heavier loads. The results we obtained indicate that with a budget of 2000 experiments it is possible to improve for up to nearly the 30% over the default configuration of Cassandra, for selected workloads, and lower budgets can anyway be used to find good quality configurations. Finally, we have analyzed the configurations obtained, and compared them with the default configuration, to understand how the better performance could be obtained.

Aside from its efficiency, irace has several other advantages: it is freely available as an R package and it is easy to use, requiring only few scripts to run, thus unburdening users and database administrators from the need of implementing complex algorithmic solutions; and it is model-free, which makes it possible to apply it to any database, benchmark or scenario with limited effort.

Several lines of research are possible to continue this work. We plan to test irace with other databases and benchmarks. In particular, we want to use it to automatically configure entire data processing pipelines of real world applications. On one hand, this will improve the performance of entire systems, not just parts of them; on the other one, we will be able the theoretical question of whether the simultaneous configuration of a single complex system can be more effective than the separate configuration of the components of the system.

Another development is the adaptation of this methodology to dynamically configure databases, periodically changing the configuration to respond to changes in the workload.

The computational cost of each evaluation is the main hurdle for a wider deployment of automatic configurators in practical applications. Thus, we want to also explore the possibility of developing cost-aware measures that can manage the configuration by estimating the cost of the experiments performed, and the potential savings entailed by the performance increase in the production environment, and autonomously determine the extent to which a tuning should be performed. Another possibility is to develop surrogate benchmarks, that can be used to estimate the performance of a candidate configuration without actually performing the evaluation.

## Supplemental Information

10.7717/peerj-cs.634/supp-1 Supplemental Information 1 Supplemental Figures and Tables.

## Additional Information and Declarations

### Data Availability

The following information was supplied regarding data availability: The Supplemental Figures and Tables are available in the Supplemental Files and at an institutional repository (http://iridia.ulb.ac.be/supp/IridiaSupp2020-014/index.html). These include additional experiments not reported in the article. The raw data and code are available at GitHub: https://github.com/MoisesSilvaM/Cassandra-Tuning.
