# Supplementary material for: Automatic configuration of the Cassandra database using irace
Source: PeerJ Comput Sci. 2021 Aug 5;7:e634. doi: 10.7717/peerj-cs.634 (PMC8356662; doi:10.7717/peerj-cs.634)
Supplement: Supplemental Information 1 [file peerj-cs-07-634-s001.pdf]

Supplementary Material for: Automatic configuration of the Cassandra database using irace

Moisés Silva-Muñoz*[1], Alberto Franzin†[1], and Hugues Bersini‡[1]

[1]IRIDIA, Université Libre de Bruxelles (ULB), Belgium

December 2020

*moises.silva.munoz@ulb.be
†afranzin@ulb.ac.be
‡bersini@ulb.ac.be

1 Finding a reliable experimental setup

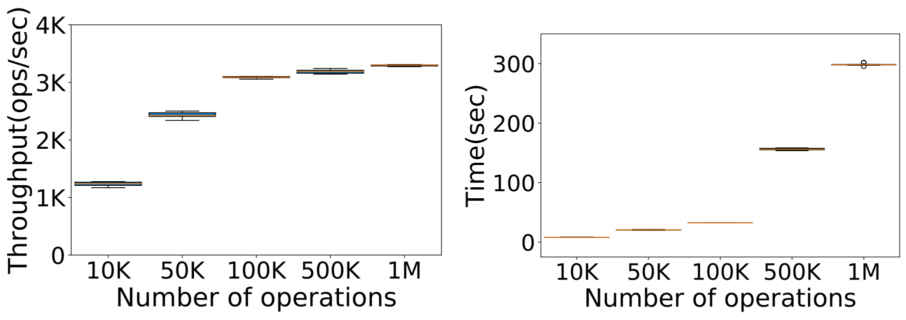

(a) Throughput (b) Time

Figure 1: Throughput obtained with 10K, 50K, 100K, 500K and 1M operations, with 100k rows of data, and the time each experiment takes. The results reported represent the performance of Cassandra measured by testing the default configuration ten times on workload A.

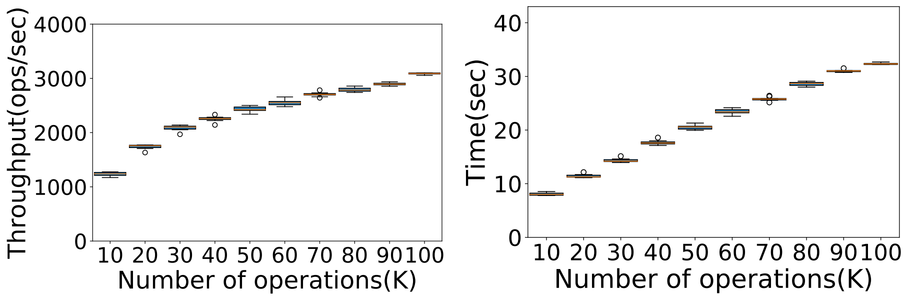

(a) Throughput (b) Time

Figure 2: Throughput obtained with 10K, 20K, 30k, 40K, 50K, 60K, 70K, 80K, 90K and 100K operations, with 100K rows of data, and the time each experiment takes. The results reported represent the performance of Cassandra measured by testing the default configuration ten times on workload A.

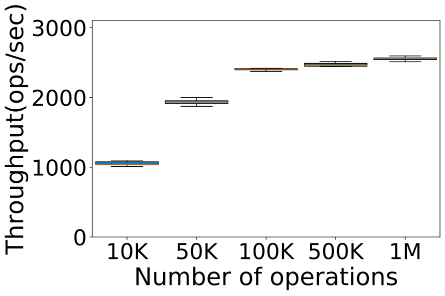

(a) Throughput

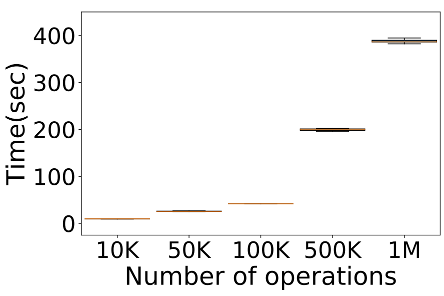

(b) Time

Figure 3: Throughput obtained with 10K, 50K, 100K, 500K and 1M operations, with 1M rows of data, and the time each experiment takes. The results reported represent the performance of Cassandra measured by testing the default configuration ten times on workload A.

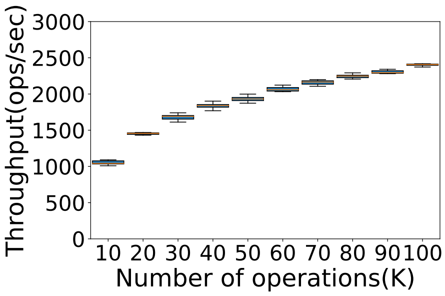

(a) Throughput

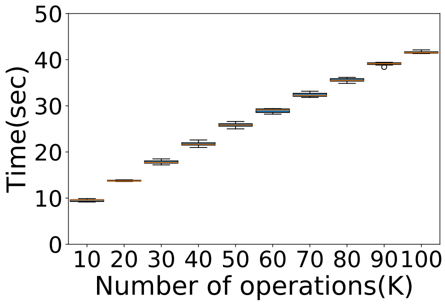

(b) Time

Figure 4: Throughput obtained with 10K, 20K, 30k, 40K, 50K, 60K, 70K, 80K, 90K and 100K operations, with 1M rows of data, and the time each experiment takes. The results reported represent the performance of Cassandra measured by testing the default configuration ten times on workload A.

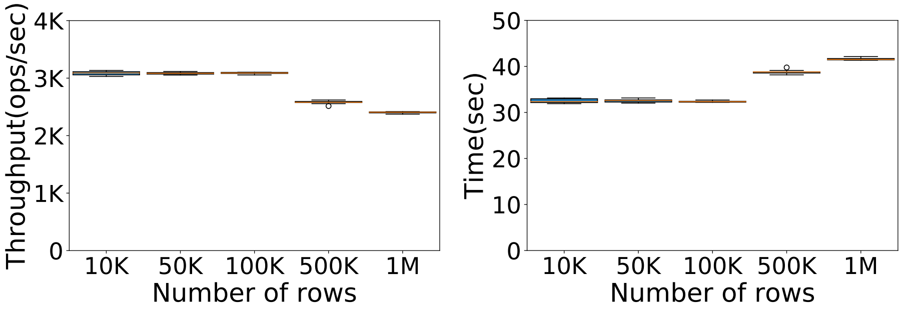

(a) Throughput (b) Time

Figure 5: Throughput obtained with 10K, 50K, 100K, 500K and 1M rows, with 100K operations per experiment, and the time each experiment takes. The results reported represent the performance of Cassandra measured by testing the default configuration ten times on workload A.

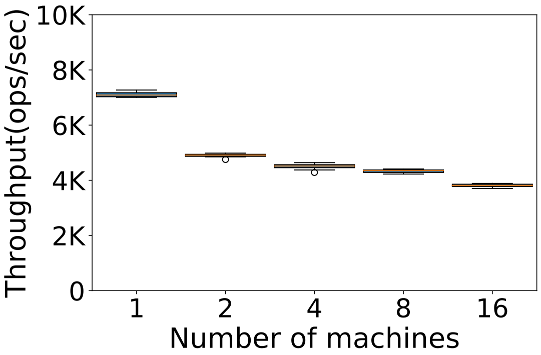

Figure 6: Throughput obtained with 100K rows and 100K operations per experiment on 1, 2, 4, 8 and 16 machines. The results reported represent the performance of Cassandra measured by testing the default configuration ten times on workload A.

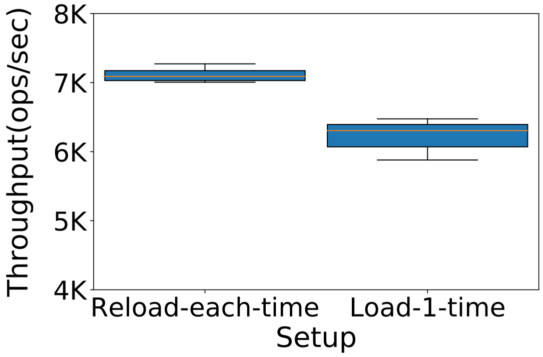

Figure 7: Throughput obtained with 100K rows and 100K operations per experiment by loading the data only once without deleting it between experiments, and destroying and reloading the data for each run. The results show the performance of Cassandra measured by testing the default configuration ten times on workload A.

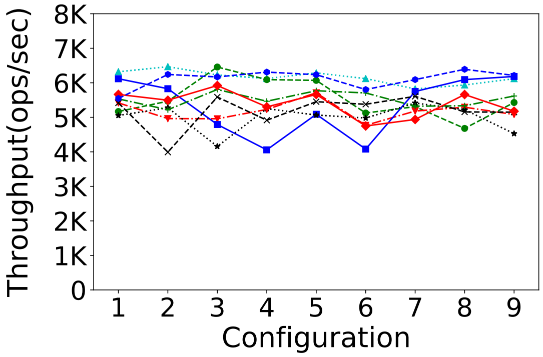

Figure 8: Throughput obtained with 100K rows and 100K operations per experiment. The results reported represent the performance of Cassandra measured by running a series of nine different setups nine times, changing the order of the configurations each run, without destroying the database after each experiment.

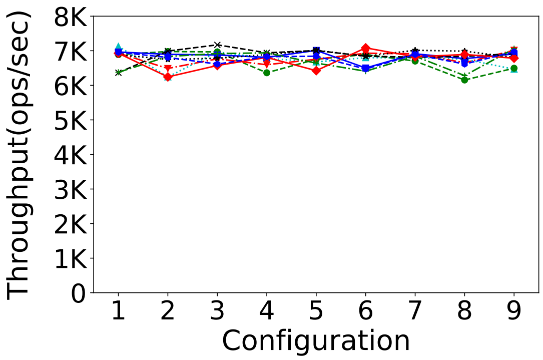

Figure 9: Throughput obtained with 100K rows and 100K operations per experiment. The results reported represent the performance of Cassandra measured by running a series of nine different setups nine times, changing the order of the configurations each run, destroying and reloading the database for each run.

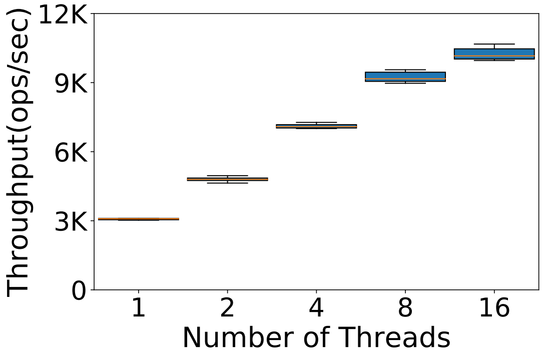

Figure 10: Throughput obtained with 100K rows and 100K operations per experiment running the experiments with 1, 2, 4, 8 and 16 threads. The results reported represent the performance of Cassandra measured by testing the default configuration ten times on workload A.

Parameter	Type	Values
**concurrent writes**	i	[8, 64]
**file cache size**	i	[256, 2048]
**memtable cleanup**	r	[0.1, 0.9]
**concurrent compact**	i	[2, 16]
**compaction strategy method**	c	(Leveled, SizeTiered)
num tokens	i	[2, 256]
concurrent reads	i	[8, 64]
replication factor	i	[2, 11]
memtable heap space	i	[1024, 3072]
memtable allocation	c	(heap_buffers, offheap_buffers, offheap_objects)
row cache size in mb	i	[0, 16]
sstable open interval	i	[0, 100]
trickle fsync	c	(True, False)
inter dc stream	i	[100, 400]
key cache size	i	[0, 200]
stream throughput	i	[100, 400]
row cache save	i	[0, 120]
column index size	i	[32, 128]
compaction throughput	i	[16, 64]
memtable offheap space	i	[1024, 3072]
commitlog segment	i	[16, 64]
mem flush writers	i	[2, 16]
index summary	i	[75, 150]

Table 1: **The 23 parameters that impact the performance of Cassandra.** Parameters of type `c` are categorical, representing alternative choices; parameters of type `i` and `r` take, respectively, integer and real values. Parameters in boldface are the most important ones for the performance of Cassandra, as determined in [1].

2 Tuning 5 parameters for all the six YCSB workloads (scenario 1

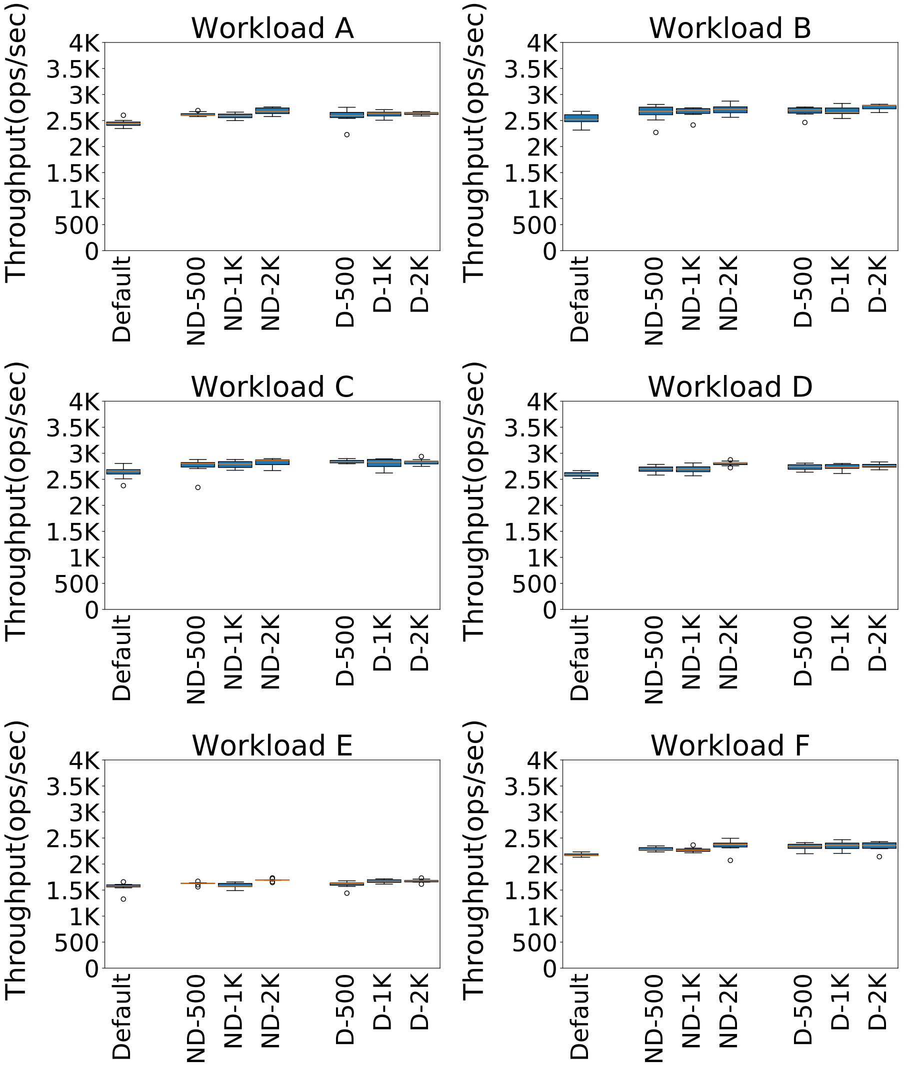

Figure 11: Throughput obtained tuning 5 parameters for all the six YCSB workloads (scenario 1). The results reported represent the throughput measured testing the final configuration over each workload, repeating each experiment ten times. The boxplots report the results obtained by, from left to right, the default configuration, irace without the default configuration (budgets of 500, 1000 and 2000 experiments), and irace with the default configuration (budgets of 500, 1000 and 2000 experiments).

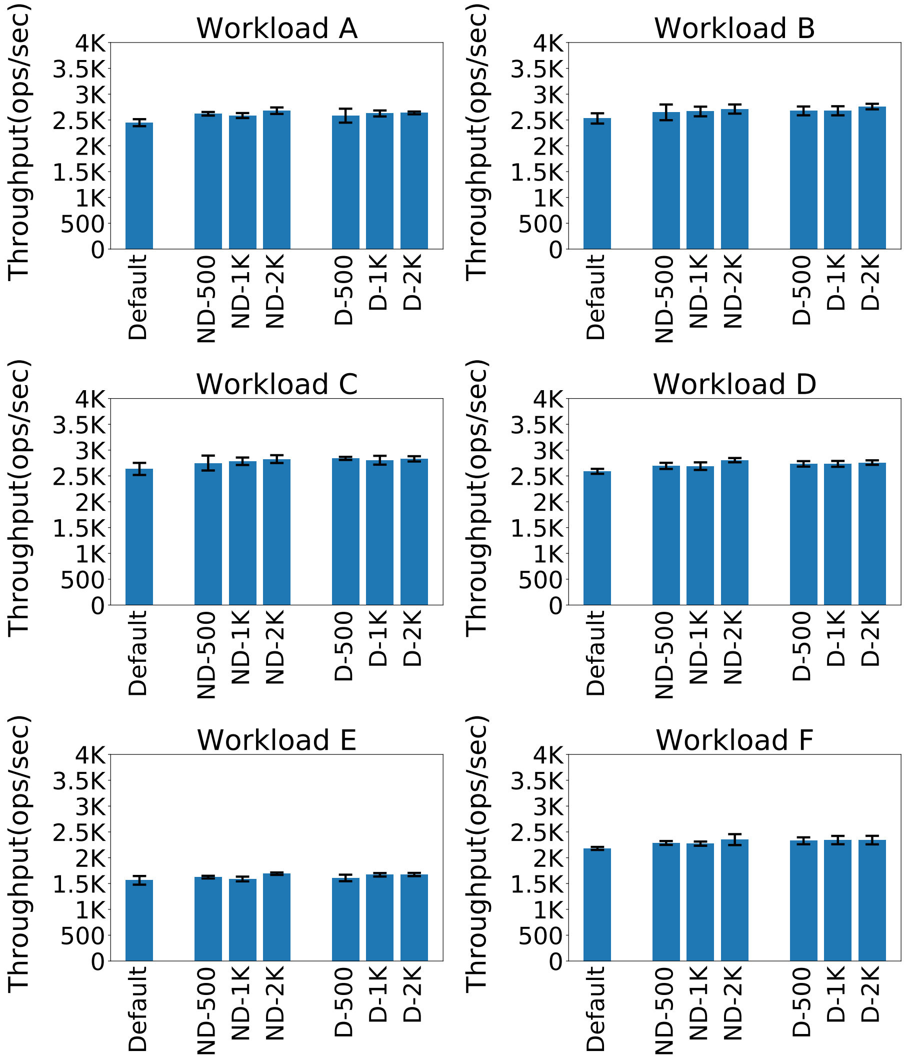

Figure 12: Throughput obtained tuning 5 parameters for all the six YCSB workloads (scenario 1). The results reported represent the throughput measured testing the final configuration over each workload, repeating each experiment ten times. The boxplots report the results obtained by, from left to right, the default configuration, irace without the default configuration (budgets of 500, 1000 and 2000 experiments), and irace with the default configuration (budgets of 500, 1000 and 2000 experiments).

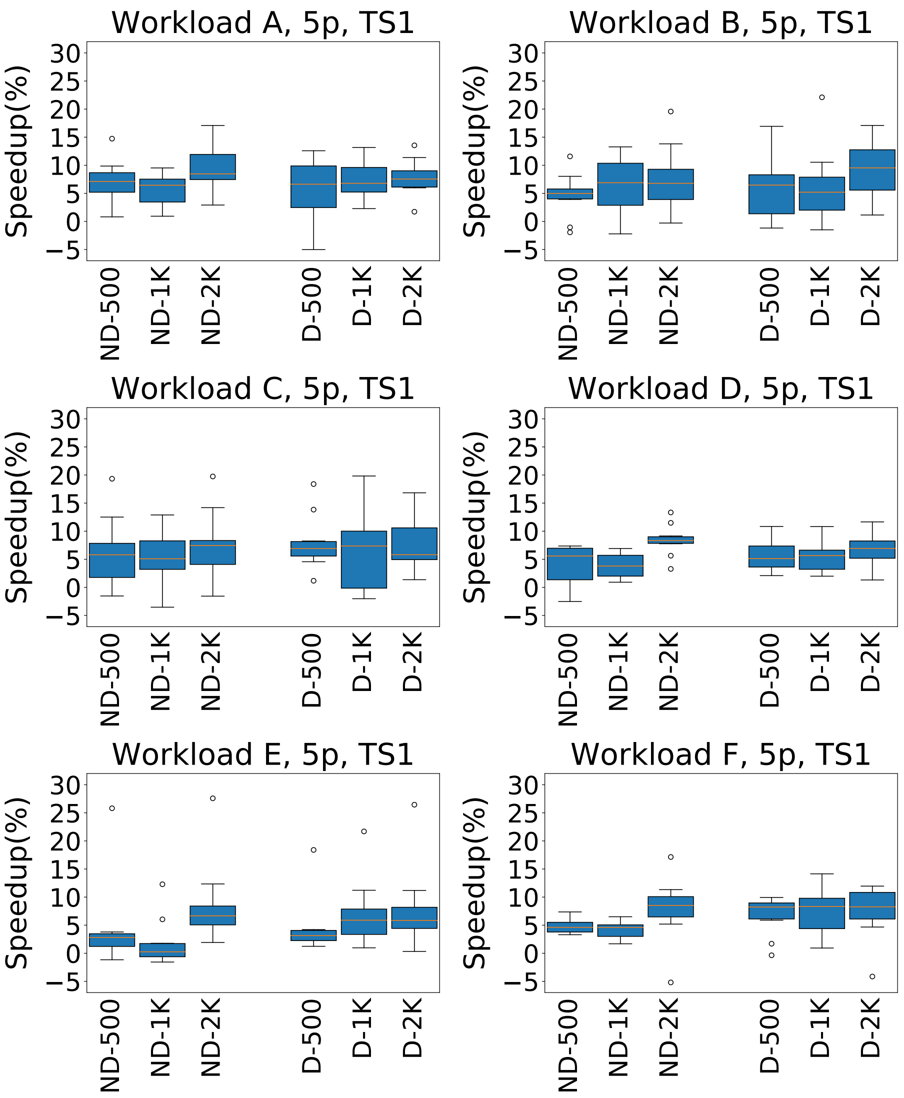

Figure 13: Speedup with respect to the default configuration obtained tuning 5 parameters for all the six YCSB workloads (scenario 1). The results reported represent the throughput measured testing the final configuration over each workload, repeating each experiment ten times. The boxplots report the speedup obtained by, from left to right, irace without the default configuration (budgets of 500, 1000 and 2000 experiments), and irace with the default configuration (budgets of 500, 1000 and 2000 experiments).

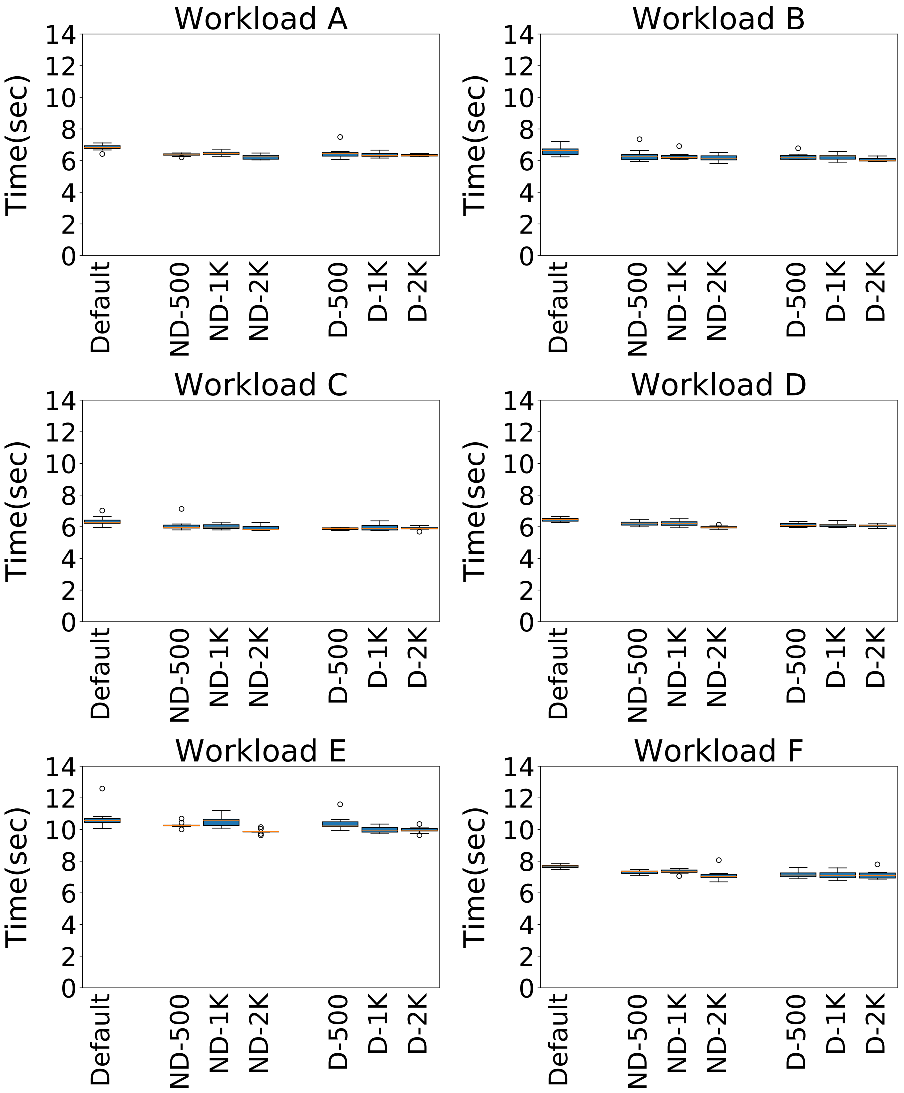

Figure 14: Time tuning 5 parameters and the 6 Workloads treated as instance in scenario 1. The results reported represent the time measured testing the final configuration over each workload, repeating each experiment ten times. The boxplots report the time obtained by, from left to right, irace without the default configuration (budgets of 500, 1000 and 2000 experiments), and irace with the default configuration (budgets of 500, 1000 and 2000 experiments).

3 Tuning 5 parameters for all the six YCSB workloads (scenario 2)

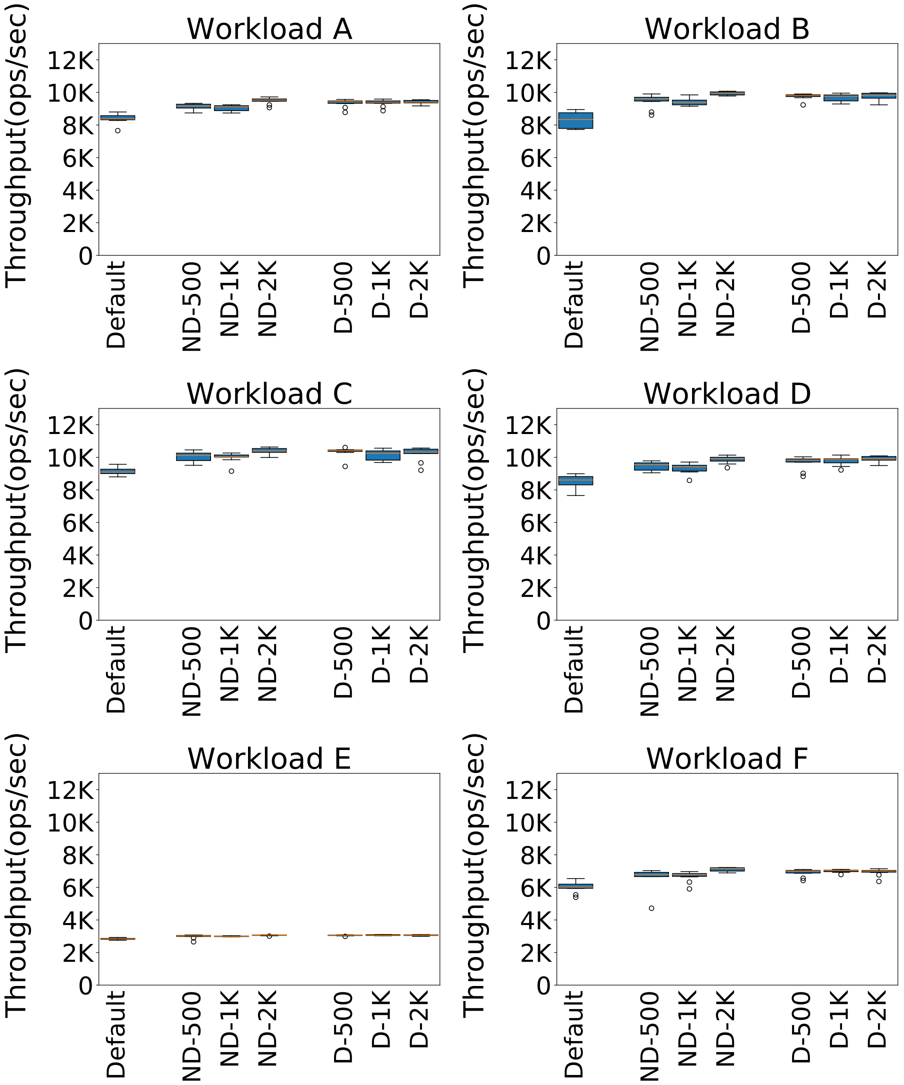

Figure 15: Throughput obtained tuning 5 parameters for all the six YCSB workloads (scenario 2). The results reported represent the throughput measured testing the final configuration over each workload, repeating each experiment ten times. The boxplots report the results obtained by, from left to right, the default configuration, irace without the default configuration (budgets of 500, 1000 and 2000 experiments), and irace with the default configuration (budgets of 500, 1000 and 2000 experiments).

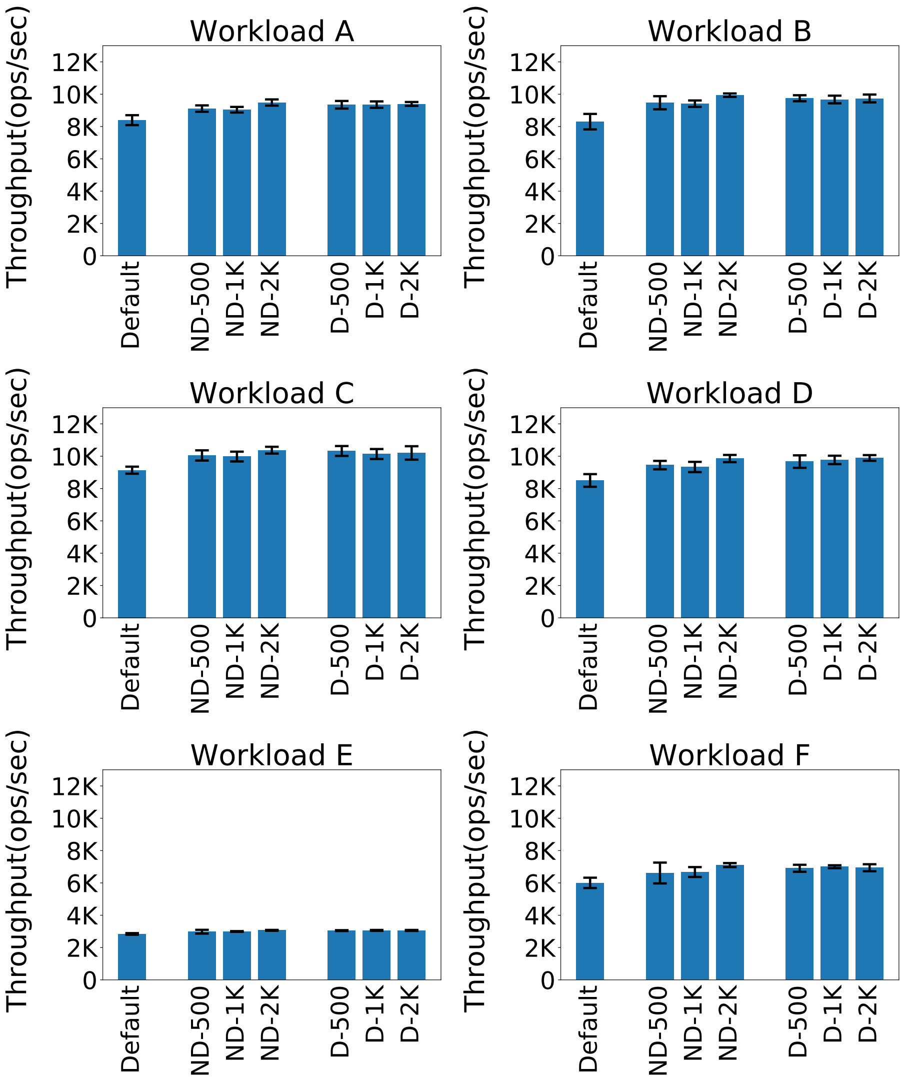

Figure 16: Throughput obtained tuning 5 parameters for all the six YCSB workloads (scenario 2). The results reported represent the throughput measured testing the final configuration over each workload, repeating each experiment ten times. The boxplots report the results obtained by, from left to right, the default configuration, irace without the default configuration (budgets of 500, 1000 and 2000 experiments), and irace with the default configuration (budgets of 500, 1000 and 2000 experiments).

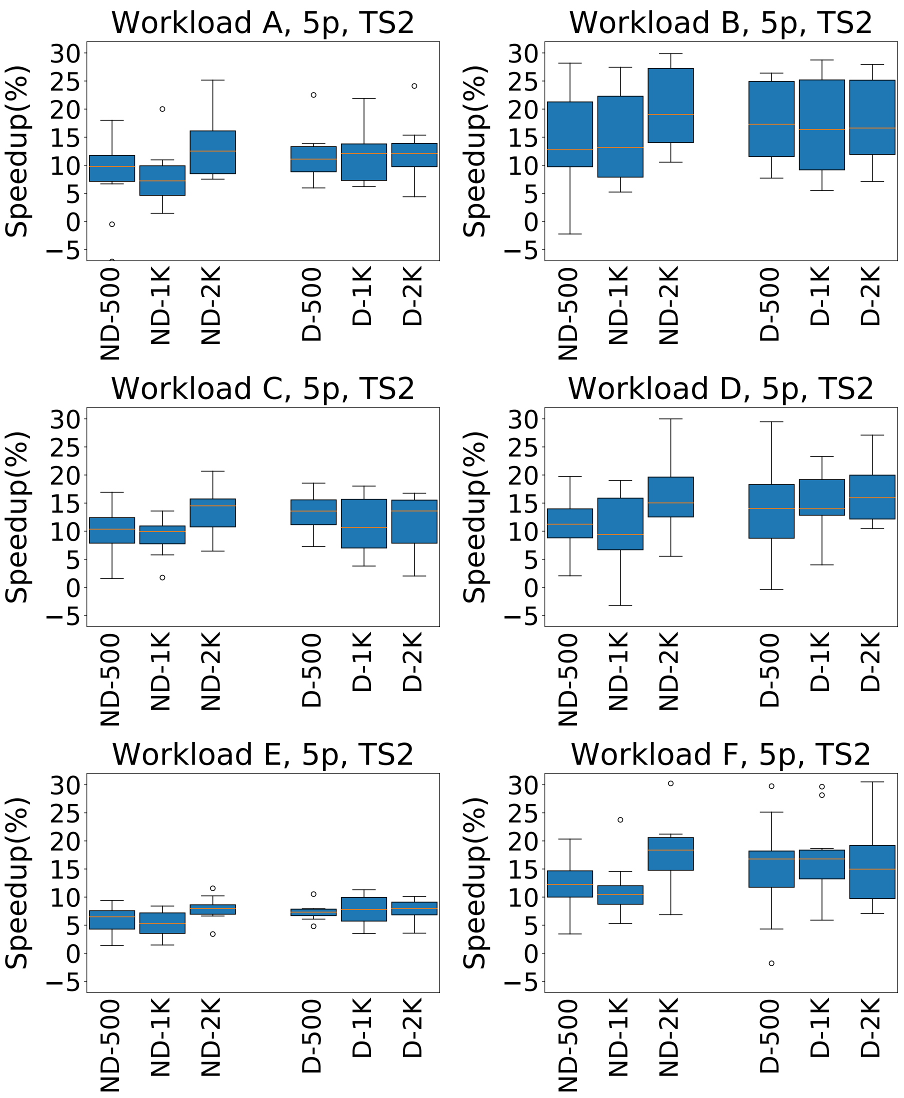

Figure 17: Speedup with respect to the default configuration obtained tuning 5 parameters for all the six YCSB workloads (scenario 2). The results reported represent the throughput measured testing the final configuration over each workload, repeating each experiment ten times. The boxplots report the speedup obtained by, from left to right, irace without the default configuration (budgets of 500, 1000 and 2000 experiments), and irace with the default configuration (budgets of 500, 1000 and 2000 experiments).

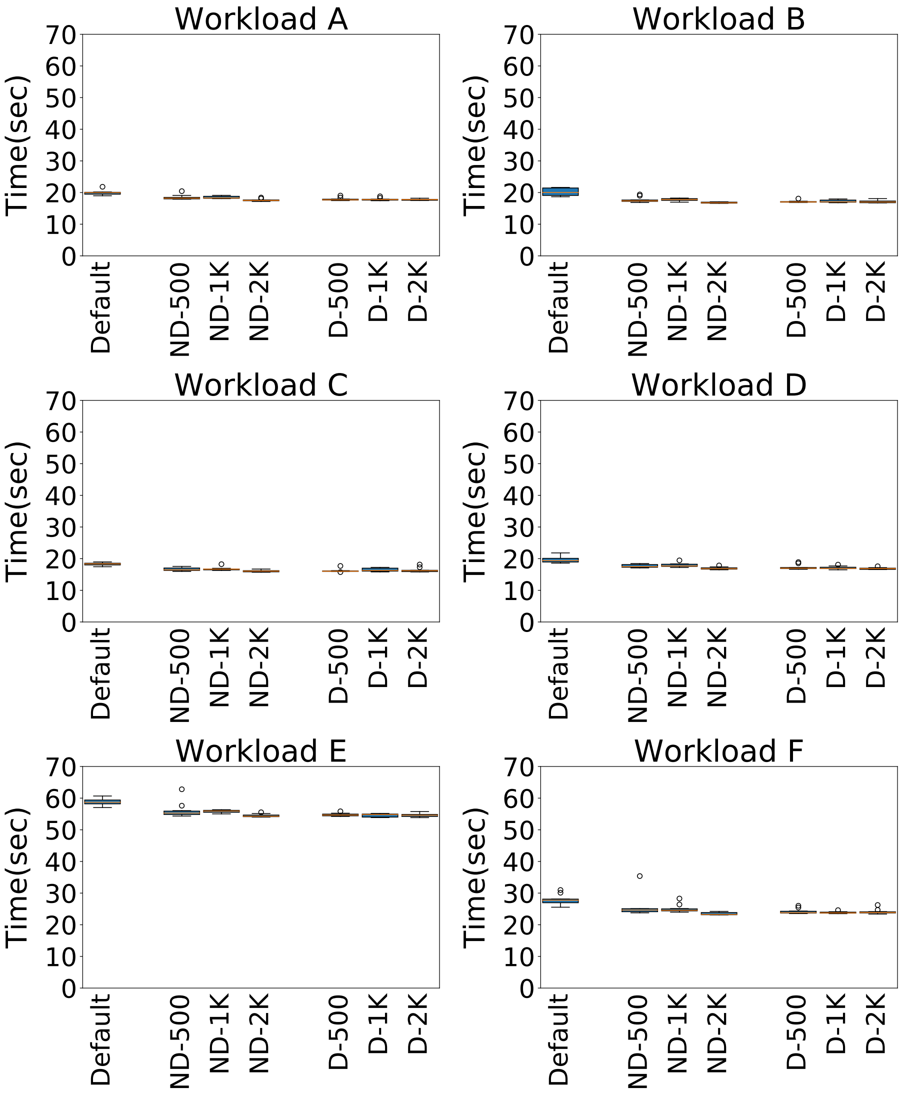

Figure 18: Time tuning 5 parameters and the 6 Workloads treated as instance in scenario 2. The results reported represent the time measured testing the final configuration over each workload, repeating each experiment ten times. The boxplots report the time obtained by, from left to right, irace without the default configuration (budgets of 500, 1000 and 2000 experiments), and irace with the default configuration (budgets of 500, 1000 and 2000 experiments).

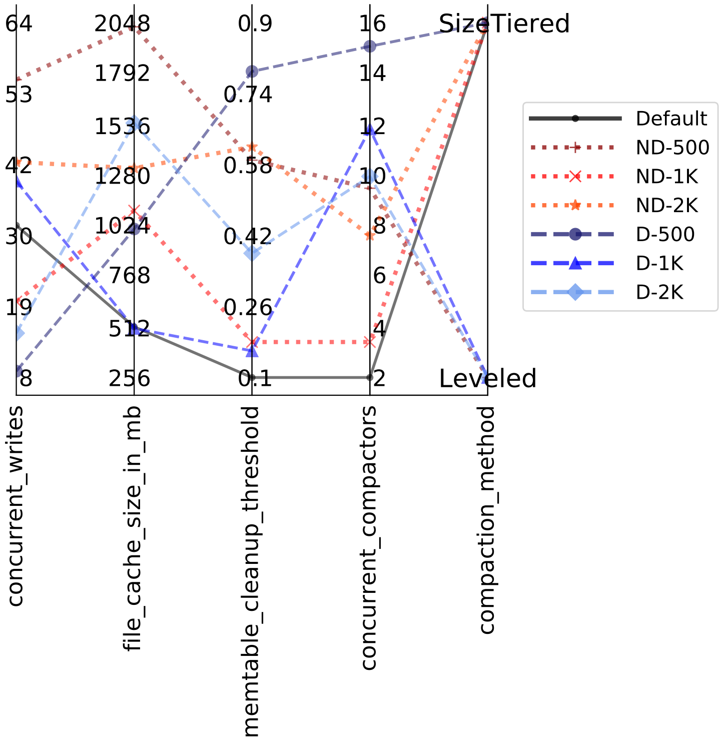

Figure 19: Parallel coordinates plot obtained tuning 5 parameters for all the 6 YCSB workloads. The reported results represent the final configuration obtained after the tuning process. The plot reports the configuration obtained by, from top to bottom, the default configuration, irace without the default configuration (budgets of 500, 1000 and 2000 experiments), and irace with the default configuration (budgets of 500, 1000 and 2000 experiments).

4 Tuning 23 parameters for all the six YCSB workloads (scenario 1)

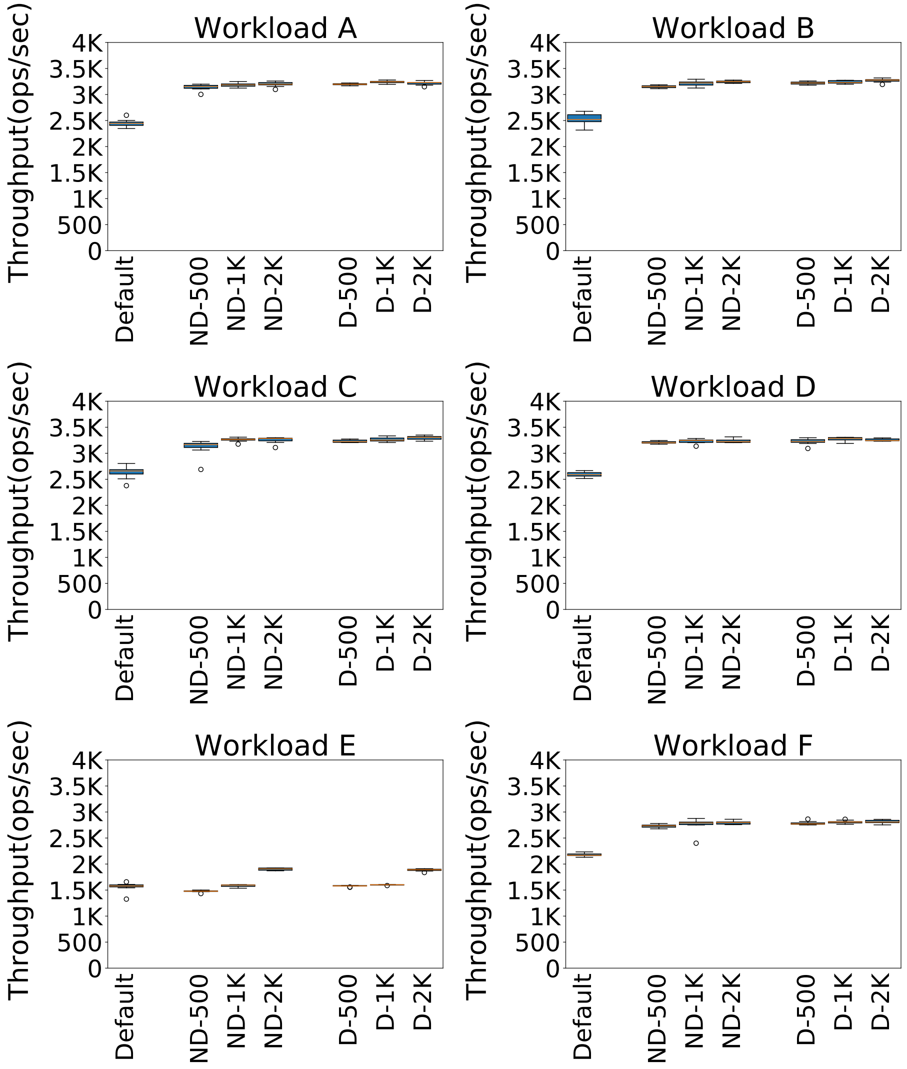

Figure 20: Throughput obtained tuning 23 parameters for all the 6 YCSB workloads (scenario 1). The results reported represent the throughput measured testing the final configuration over each workload, repeating each experiment ten times. The boxplots report the results obtained by, from left to right, the default configuration, irace without the default configuration (budgets of 500, 1000 and 2000 experiments), and irace with the default configuration (budgets of 500, 1000 and 2000 experiments).

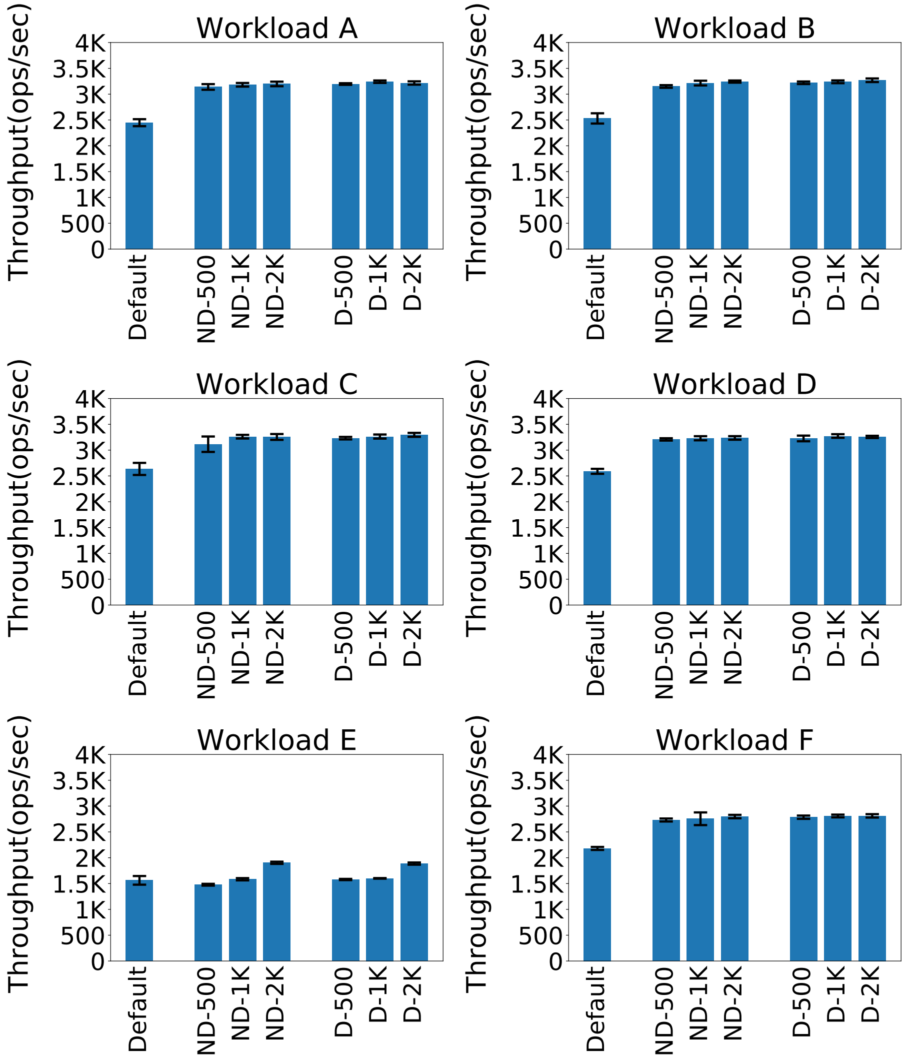

Figure 21: Throughput obtained tuning 23 parameters for all the 6 YCSB workloads (scenario 1). The results reported represent the throughput measured testing the final configuration over each workload, repeating each experiment ten times. The boxplots report the results obtained by, from left to right, the default configuration, irace without the default configuration (budgets of 500, 1000 and 2000 experiments), and irace with the default configuration (budgets of 500, 1000 and 2000 experiments).

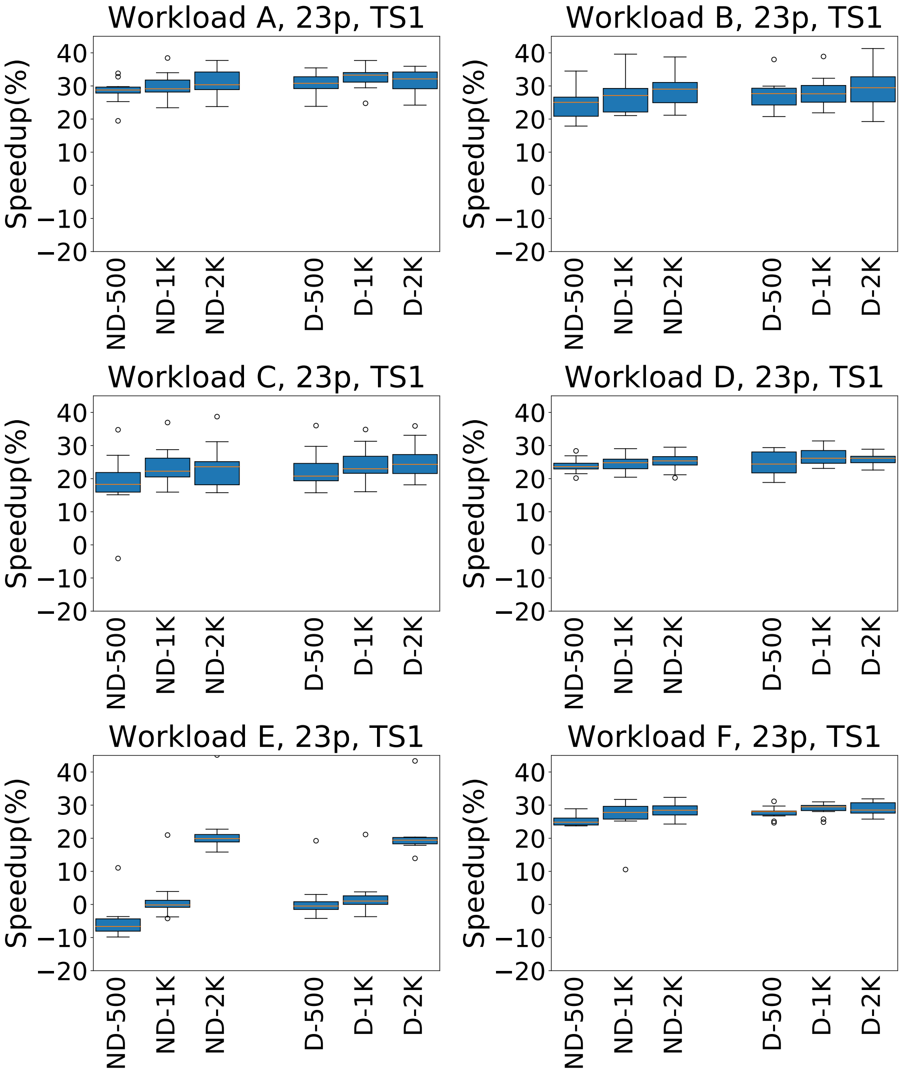

Figure 22: Speedup with respect to the default configuration obtained tuning 23 parameters for all the 6 YCSB workloads (scenario 1). The results reported represent the throughput measured testing the final configuration over each workload, repeating each experiment ten times. The boxplots report the speedup obtained by, from left to right, irace without the default configuration (budgets of 500, 1000 and 2000 experiments), and irace with the default configuration (budgets of 500, 1000 and 2000 experiments).

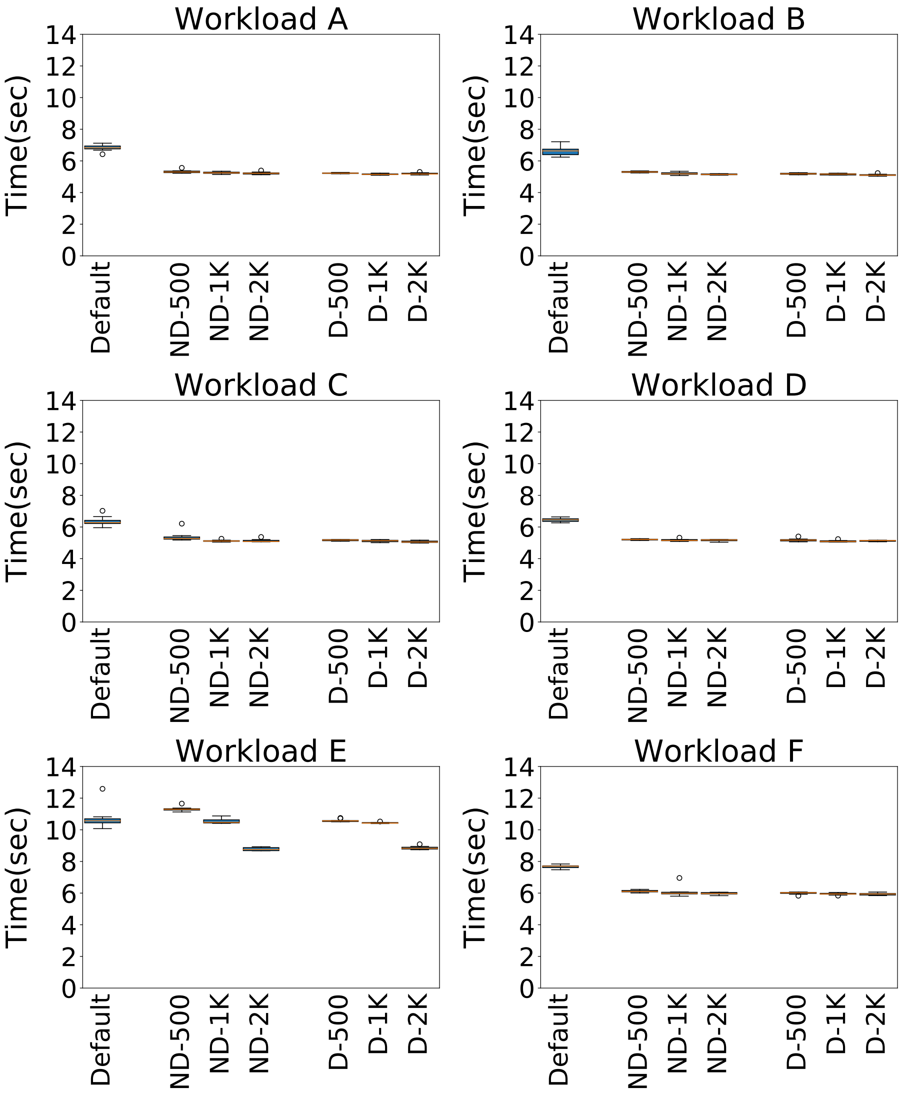

Figure 23: Time tuning 23 parameters and the 6 Workloads treated as instance in scenario 1. The results reported represent the time measured testing the final configuration over each workload, repeating each experiment ten times. The boxplots report the time obtained by, from left to right, irace without the default configuration (budgets of 500, 1000 and 2000 experiments), and irace with the default configuration (budgets of 500, 1000 and 2000 experiments).

5 Tuning 23 parameters for all the six YCSB workloads (scenario 2)

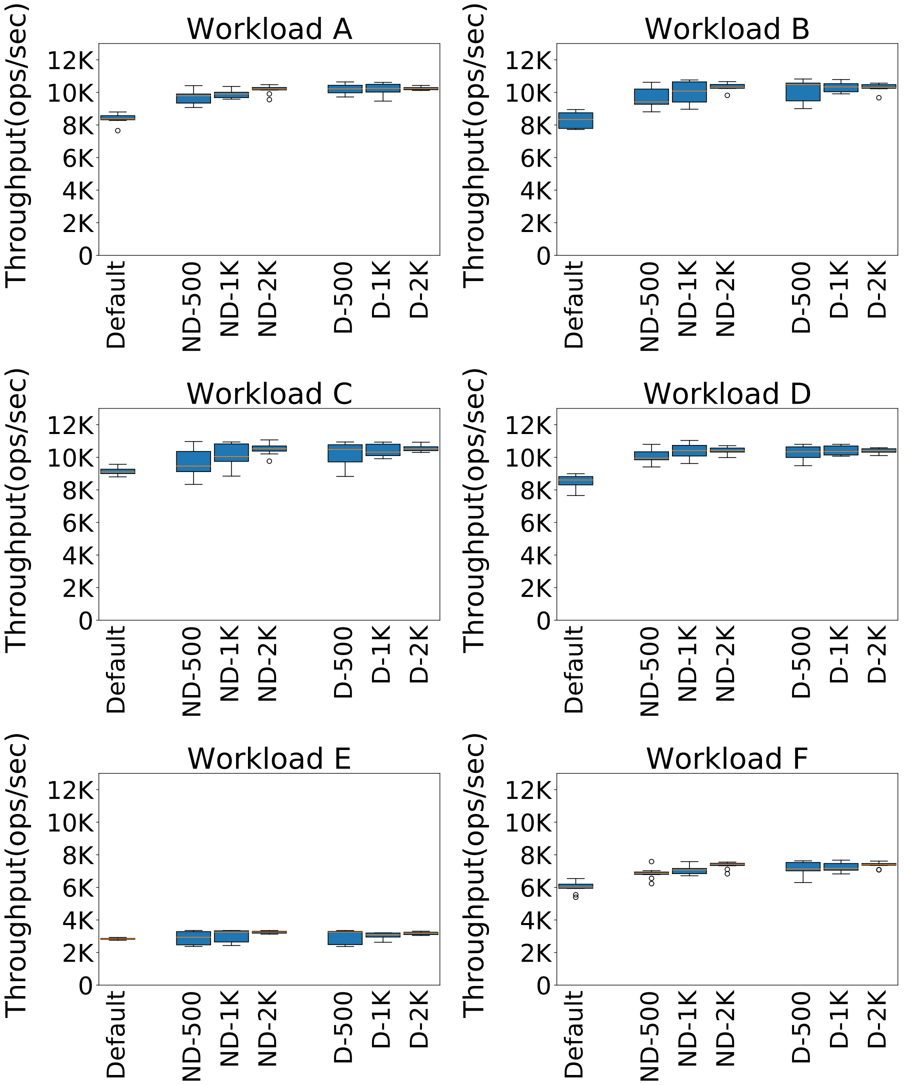

Figure 24: Throughput obtained tuning 23 parameters for all the 6 YCSB workloads (scenario 2). The results reported represent the throughput measured testing the final configuration over each workload, repeating each experiment ten times. The boxplots report the results obtained by, from left to right, the default configuration, irace without the default configuration (budgets of 500, 1000 and 2000 experiments), and irace with the default configuration (budgets of 500, 1000 and 2000 experiments).

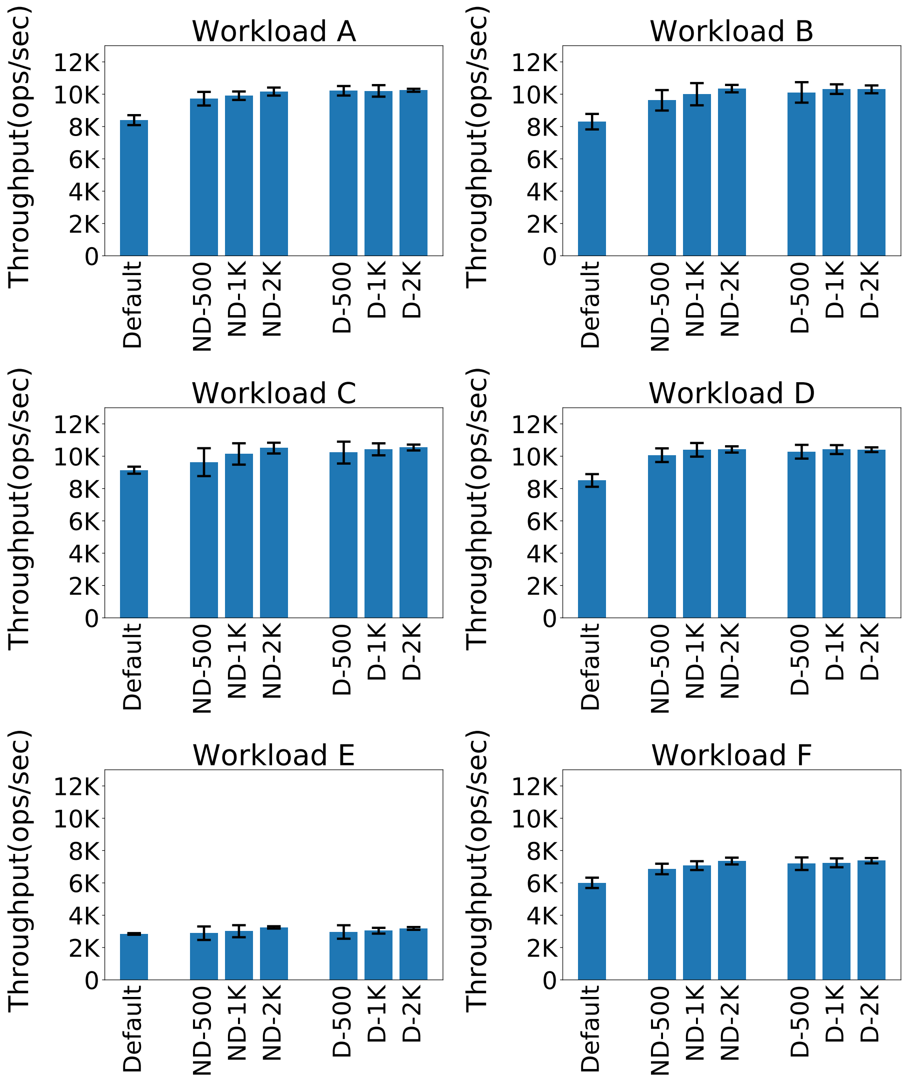

Figure 25: Throughput obtained tuning 23 parameters for all the 6 YCSB workloads (scenario 2). The results reported represent the throughput measured testing the final configuration over each workload, repeating each experiment ten times. The boxplots report the results obtained by, from left to right, the default configuration, irace without the default configuration (budgets of 500, 1000 and 2000 experiments), and irace with the default configuration (budgets of 500, 1000 and 2000 experiments).

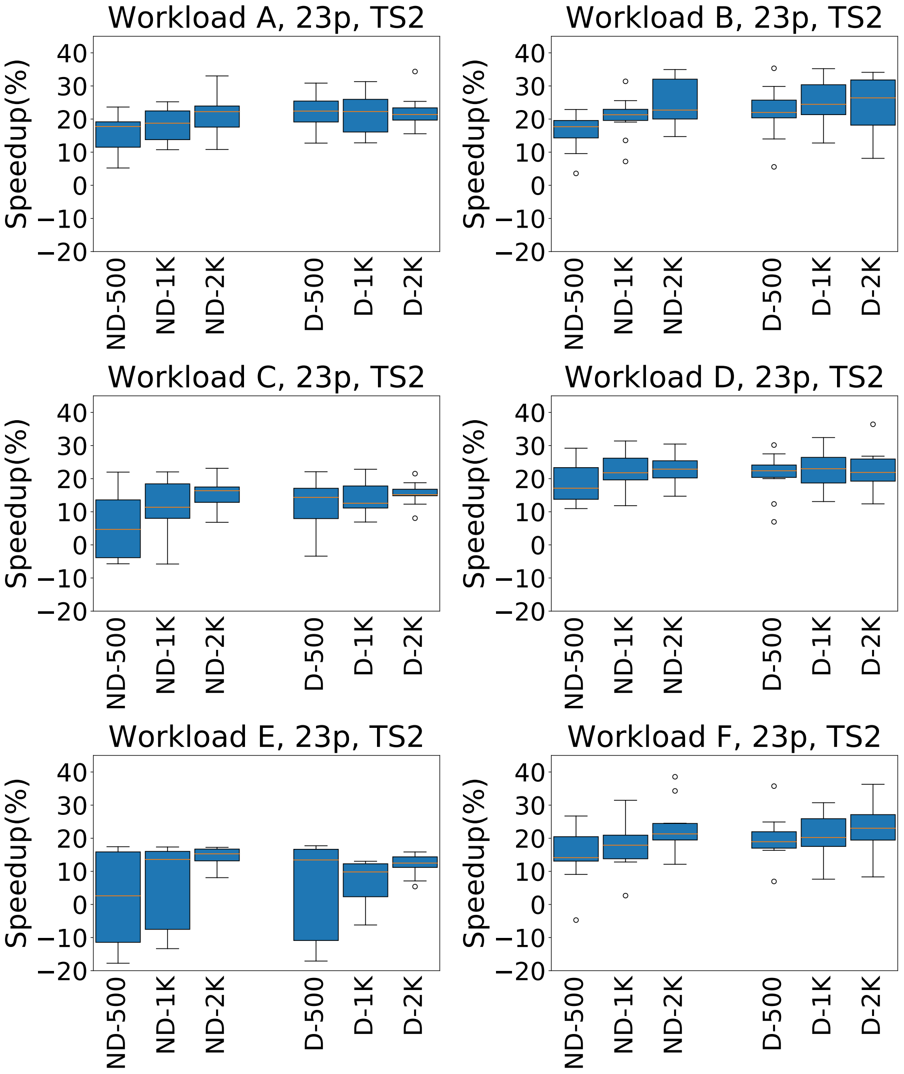

Figure 26: Speedup with respect to the default configuration obtained tuning 23 parameters for all the 6 YCSB workloads (scenario 2). The results reported represent the throughput measured testing the final configuration over each workload, repeating each experiment ten times. The boxplots report the speedup obtained by, from left to right, irace without the default configuration (budgets of 500, 1000 and 2000 experiments), and irace with the default configuration (budgets of 500, 1000 and 2000 experiments).

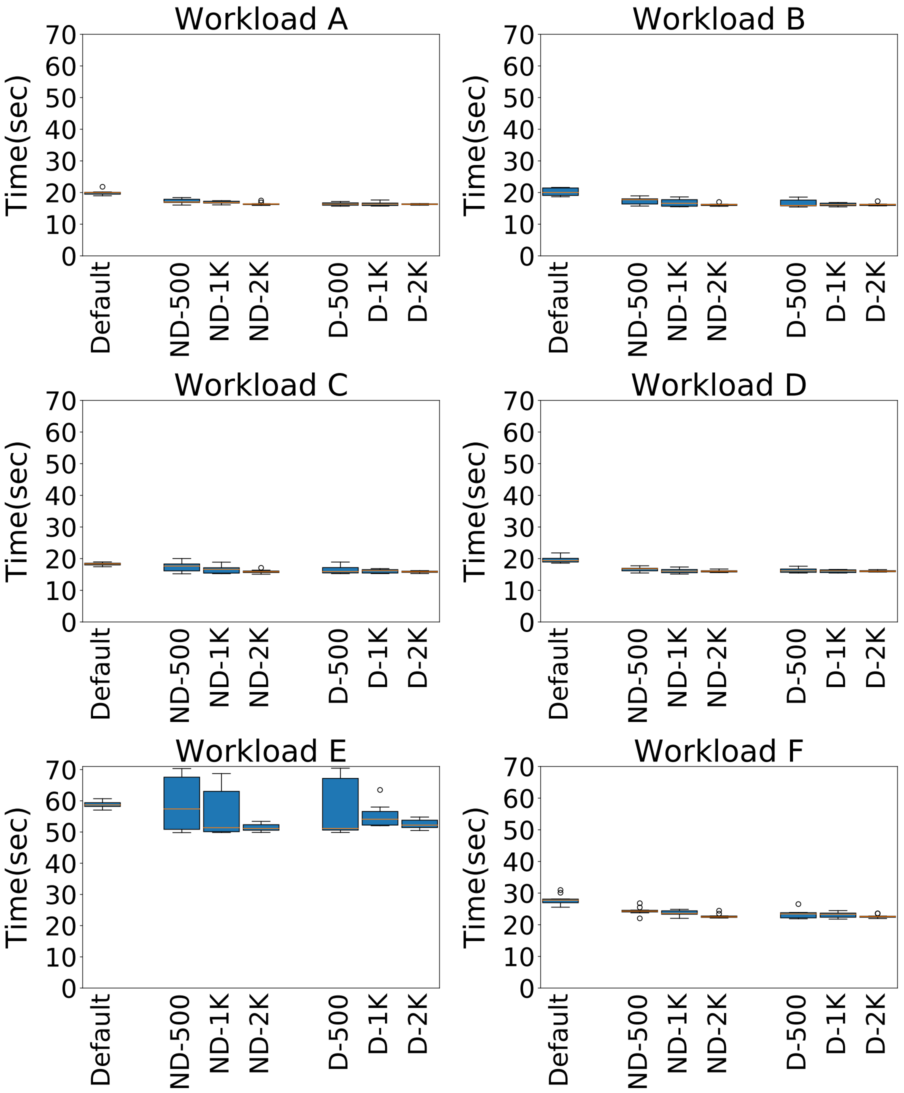

Figure 27: Time tuning 23 parameters and the 6 Workloads treated as instance in scenario 2. The results reported represent the time measured testing the final configuration over each workload, repeating each experiment ten times. The boxplots report the time obtained by, from left to right, irace without the default configuration (budgets of 500, 1000 and 2000 experiments), and irace with the default configuration (budgets of 500, 1000 and 2000 experiments).

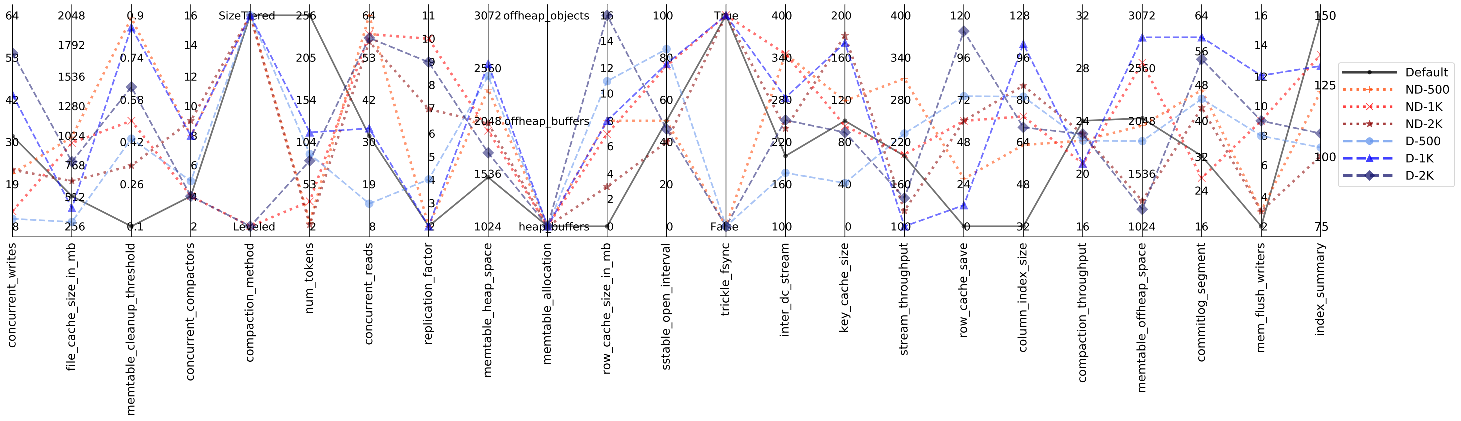

Figure 28: Parallel coordinates plot obtained tuning 23 parameters for all the 6 YCSB workloads. The reported results represent the final configuration obtained after the tuning process. The plot reports the configuration obtained by, from top to bottom, the default configuration, irace without the default configuration (budgets of 500, 1000 and 2000 experiments), and irace with the default configuration (budgets of 500, 1000 and 2000 experiments).

6 Tuning 5 parameters for workload A (scenario 1)

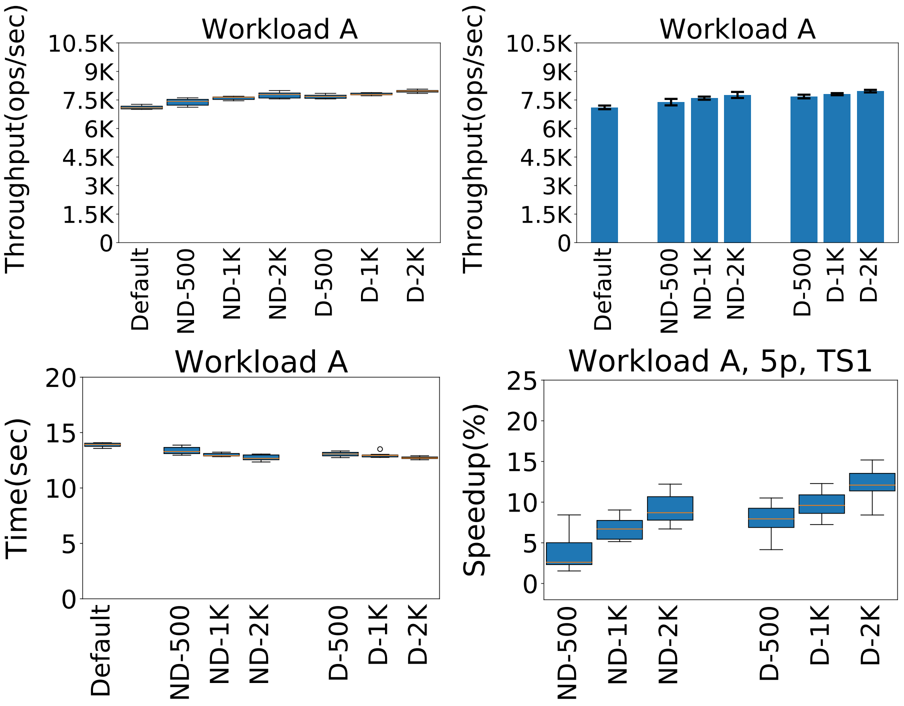

Figure 29: Results obtained when tuning the YCSB workload A in test scenario 1 tuning 5 parameters. The results reported the boxplot and the histogram of the throughput in addition to the boxplot of the speedup and the time measured the final configurations, repeating each experiment ten times. The boxplots report the results obtained by, from left to right, the default configuration, irace without the default configuration (budgets of 500, 1000 and 2000 experiments), and irace with the default configuration (budgets of 500, 1000 and 2000 experiments). The speedup is computed with respect to the default configuration.

7 Tuning 5 parameters for workload A (scenario 2)

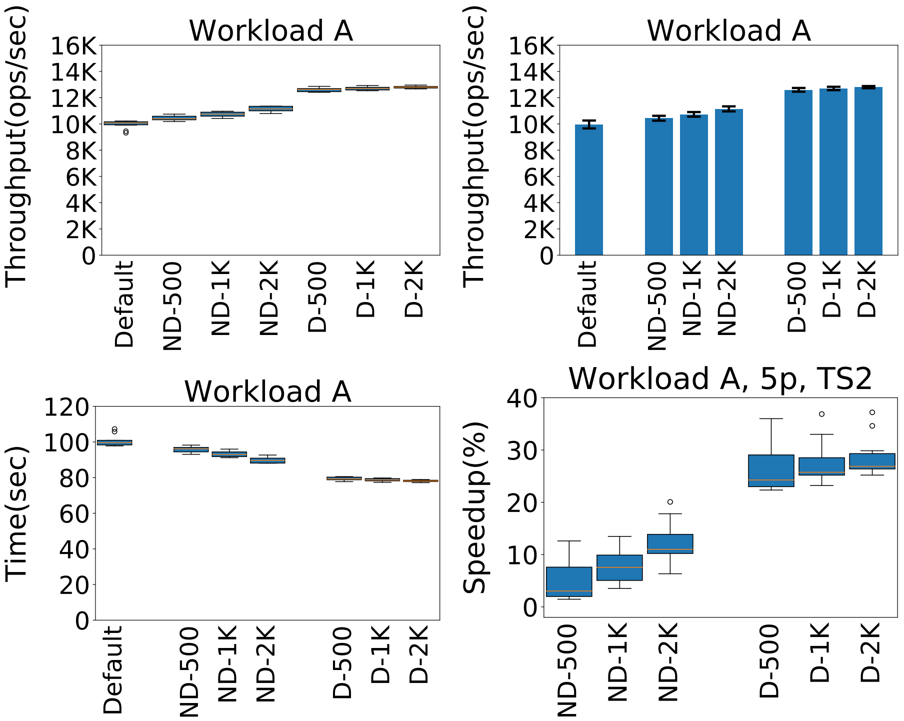

Figure 30: Results obtained when tuning the YCSB workload A in scenario 2 tuning 5 parameters. The results reported the boxplot and the histogram of the throughput in addition to the boxplot of the speedup and the time measured the final configurations, repeating each experiment ten times. The boxplots report the results obtained by, from left to right, the default configuration, irace without the default configuration (budgets of 500, 1000 and 2000 experiments), and irace with the default configuration (budgets of 500, 1000 and 2000 experiments). The speedup is computed with respect to the default configuration.

8 Parallel coordinates plot obtained tuning 5 parameters for workload A

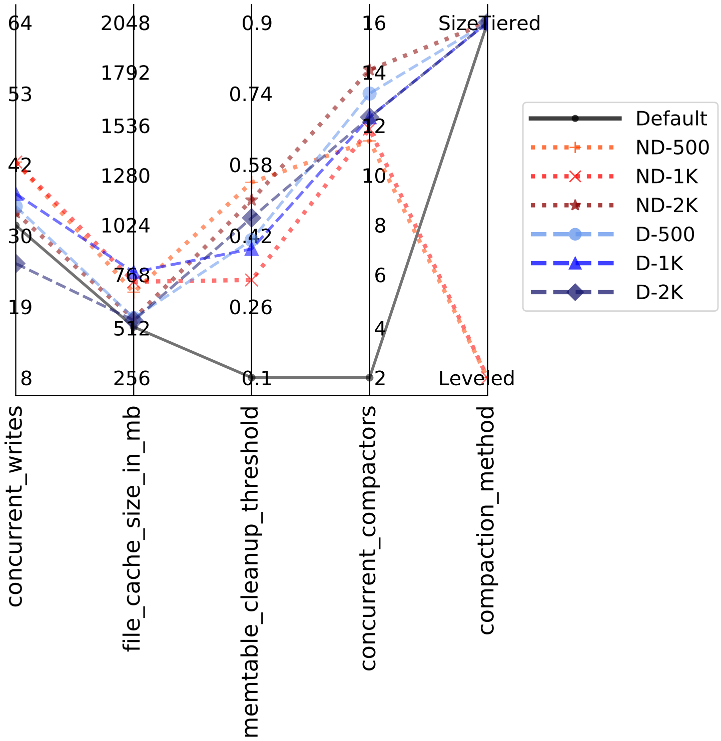

Figure 31: Parallel coordinates plot obtained tuning 5 parameters for workload A. The reported results represent the final configuration obtained after the tuning process. The plot reports the configuration obtained by, from top to bottom, the default configuration, irace without the default configuration (budgets of 500, 1000 and 2000 experiments), and irace with the default configuration (budgets of 500, 1000 and 2000 experiments).

9 Tuning 23 parameters for workload A (scenario 1)

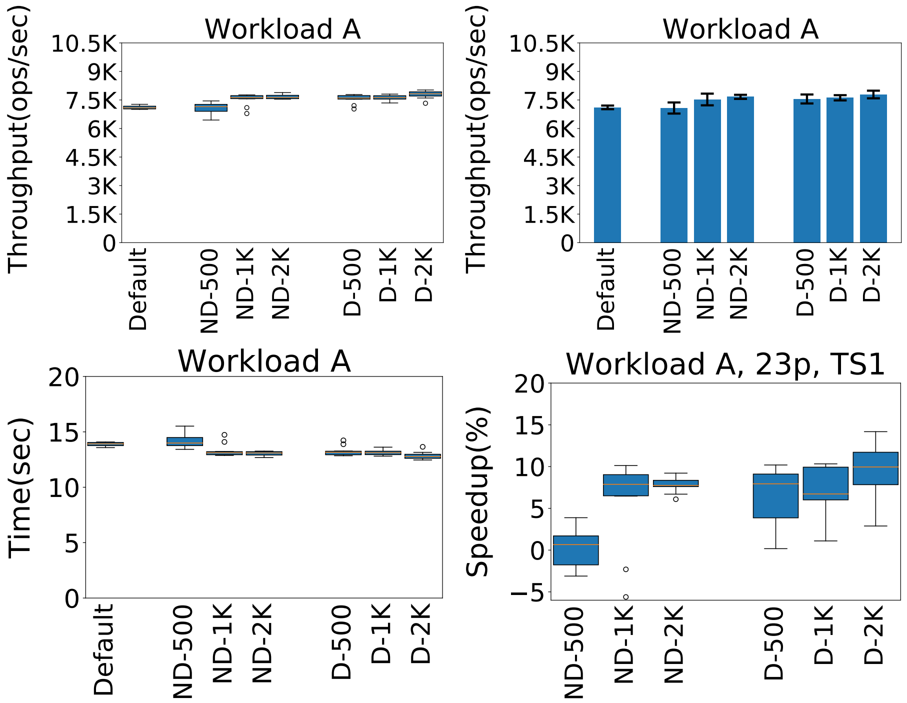

Figure 32: Results obtained when tuning the YCSB workload A with 23 parameters (scenario 1). The results reported the boxplot and the histogram of the throughput in addition to the boxplot of the speedup and the boxplot of the time measured in the final configurations in scenario 1, repeating each experiment ten times. The boxplots report the results obtained by, from left to right, the default configuration, irace without the default configuration (budgets of 500, 1000 and 2000 experiments), and irace with the default configuration (budgets of 500, 1000 and 2000 experiments). The speedup is computed with respect to the default configuration.

10 Tuning 23 parameters for workload A (scenario 2)

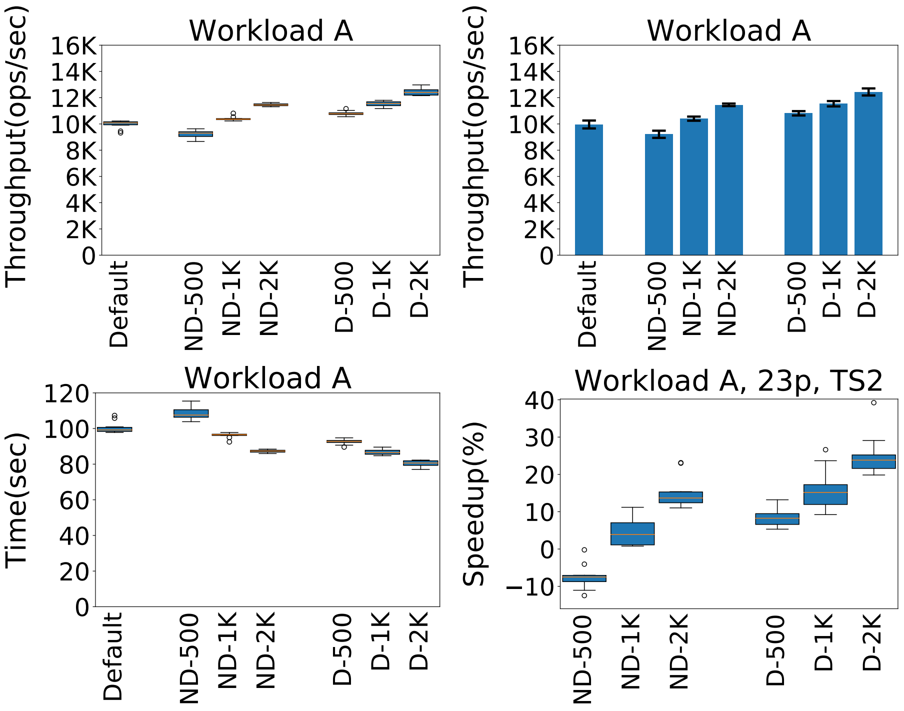

Figure 33: Results obtained when tuning the YCSB workload A with 23 parameters (scenario 2). The results reported the boxplot and the histogram of the throughput in addition to the boxplot of the speedup and the time measured the in the final configurations in scenario 2, repeating each experiment ten times. The boxplots report the results obtained by, from left to right, the default configuration, irace without the default configuration (budgets of 500, 1000 and 2000 experiments), and irace with the default configuration (budgets of 500, 1000 and 2000 experiments). The speedup is computed with respect to the default configuration.

11 Parallel coordinates plot obtained tuning 23 parameters for workload A

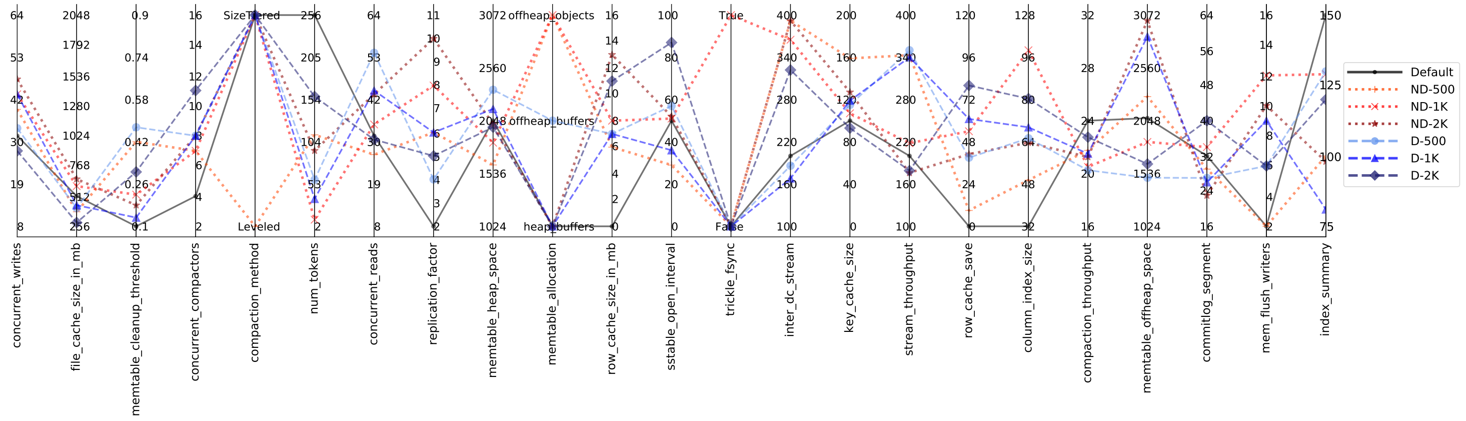

Figure 34: Parallel coordinates plot obtained tuning 23 parameters for workload A. The reported results represent the final configuration obtained after the tuning process. The plot reports the configuration obtained by, from top to bottom, the default configuration, irace without the default configuration (budgets of 500, 1000 and 2000 experiments), and irace with the default configuration (budgets of 500, 1000 and 2000 experiments).

12 Tuning 5 parameters for workload E (scenario 1)

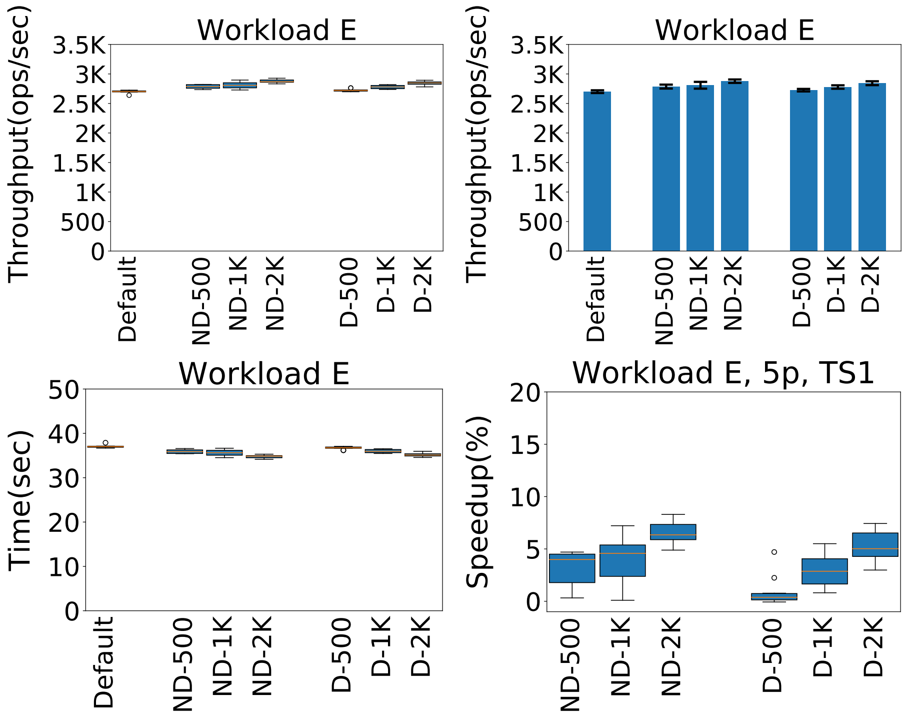

Figure 35: Results obtained when tuning the YCSB workload E with 5 parameters (scenario 1). The results reported the boxplot and the histogram of the throughput in addition to the boxplot of the speedup and the boxplot of the time measured in the final configurations in scenario 1, repeating each experiment ten times. The boxplots report the results obtained by, from left to right, the default configuration, irace without the default configuration (budgets of 500, 1000 and 2000 experiments), and irace with the default configuration (budgets of 500, 1000 and 2000 experiments). The speedup is computed with respect to the default configuration.

13 Tuning 5 parameters for workload E (scenario 2)

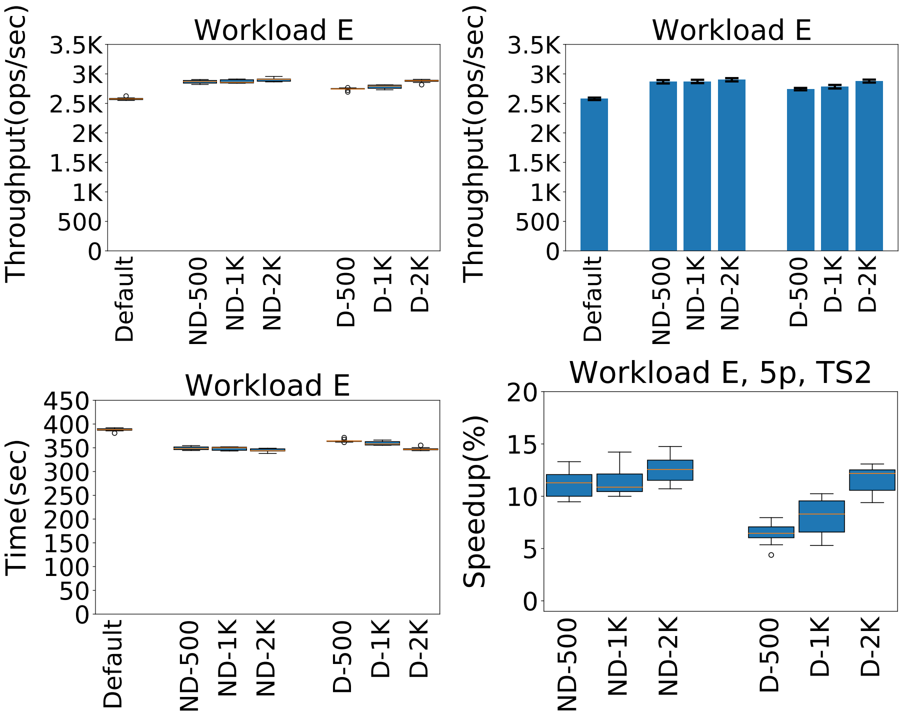

Figure 36: Results obtained when tuning the YCSB workload E with 5 parameters (scenario 2). The results reported the boxplot and the histogram of the throughput in addition to the boxplot of the speedup and the time measured the in the final configurations in scenario 2, repeating each experiment ten times. The boxplots report the results obtained by, from left to right, the default configuration, irace without the default configuration (budgets of 500, 1000 and 2000 experiments), and irace with the default configuration (budgets of 500, 1000 and 2000 experiments). The speedup is computed with respect to the default configuration.

14 Parallel coordinates plot obtained tuning 5 parameters for workload E

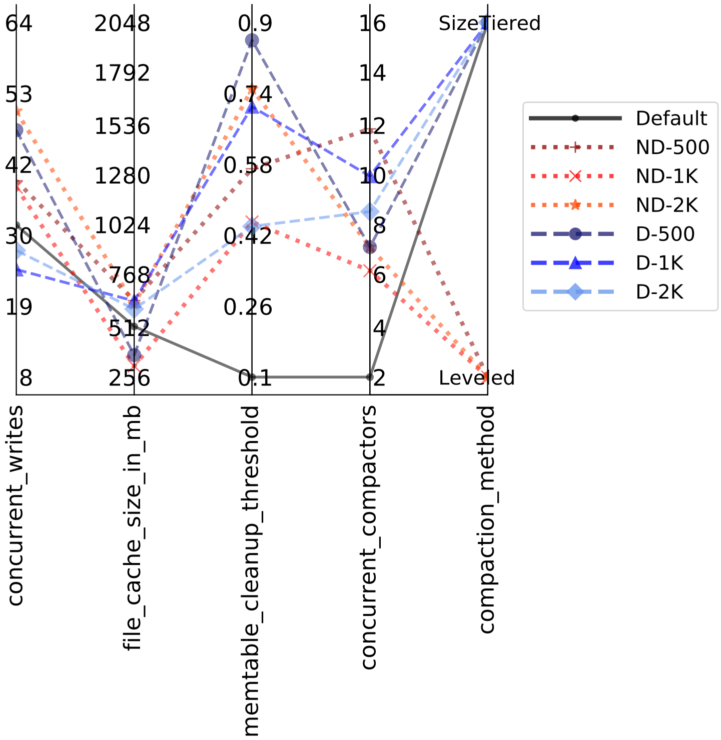

Figure 37: Parallel coordinates plot obtained tuning 5 parameters for workload E. The reported results represent the final configuration obtained after the tuning process. The plot reports the configuration obtained by, from top to bottom, the default configuration, irace without the default configuration (budgets of 500, 1000 and 2000 experiments), and irace with the default configuration (budgets of 500, 1000 and 2000 experiments).

15 Tuning 23 parameters for workload E (scenario 1)

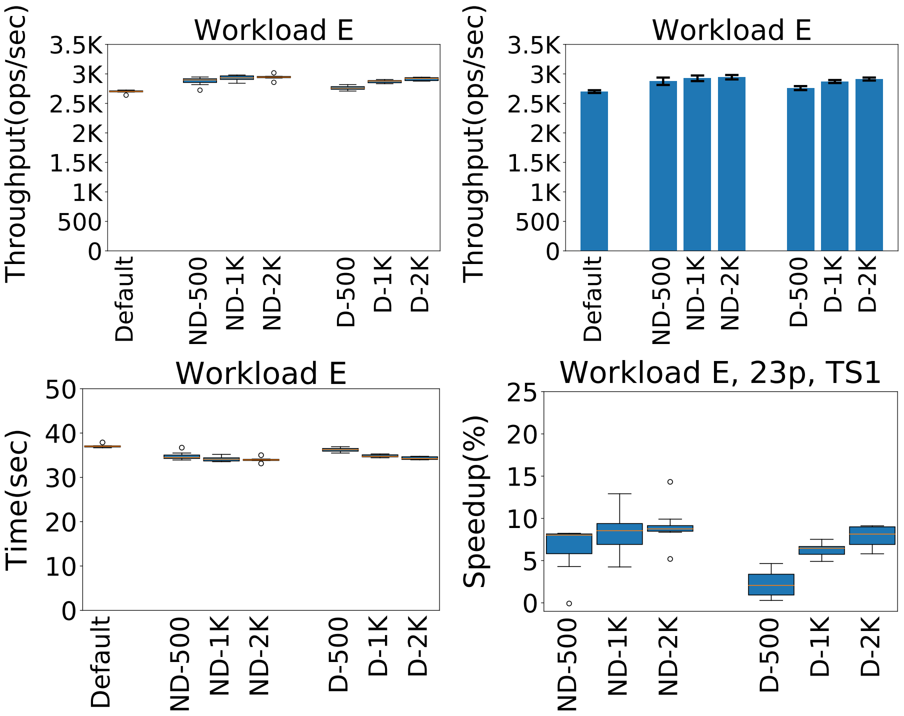

Figure 38: Results obtained when tuning the YCSB workload E with 23 parameters (scenario 1). The results reported the boxplot and the histogram of the throughput in addition to the boxplot of the speedup and the boxplot of the time measured in the final configurations in scenario 1, repeating each experiment ten times. The boxplots report the results obtained by, from left to right, the default configuration, irace without the default configuration (budgets of 500, 1000 and 2000 experiments), and irace with the default configuration (budgets of 500, 1000 and 2000 experiments). The speedup is computed with respect to the default configuration.

16 Tuning 23 parameters for workload E (scenario 2)

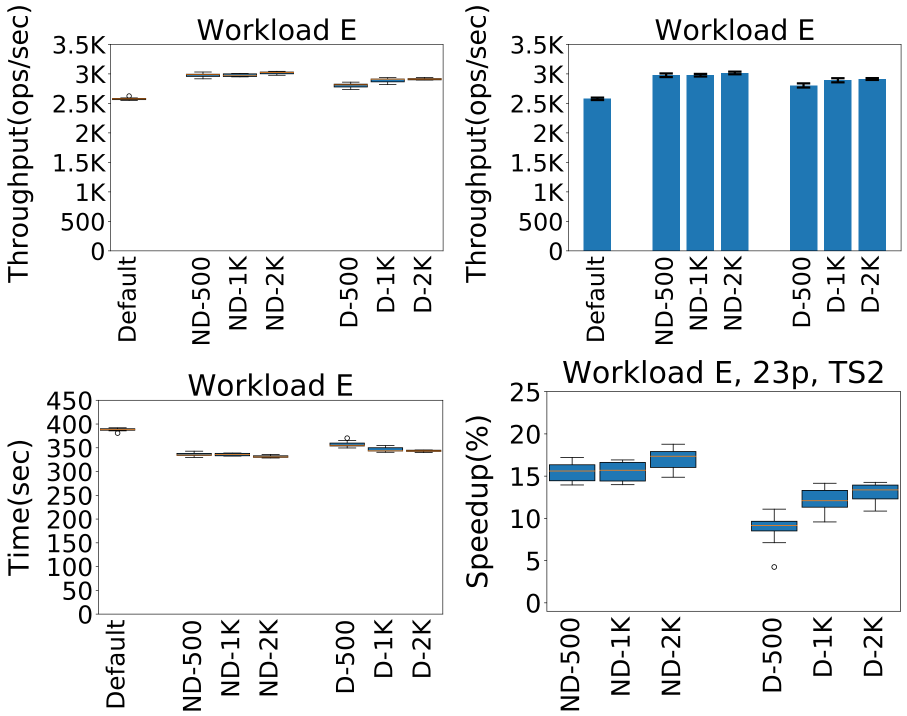

Figure 39: Results obtained when tuning the YCSB workload E with 23 parameters (scenario 2). The results reported the boxplot and the histogram of the throughput in addition to the boxplot of the speedup and the time measured the in the final configurations in scenario 2, repeating each experiment ten times. The boxplots report the results obtained by, from left to right, the default configuration, irace without the default configuration (budgets of 500, 1000 and 2000 experiments), and irace with the default configuration (budgets of 500, 1000 and 2000 experiments). The speedup is computed with respect to the default configuration.

17 Parallel coordinates plot obtained tuning 23 parameters for workload E

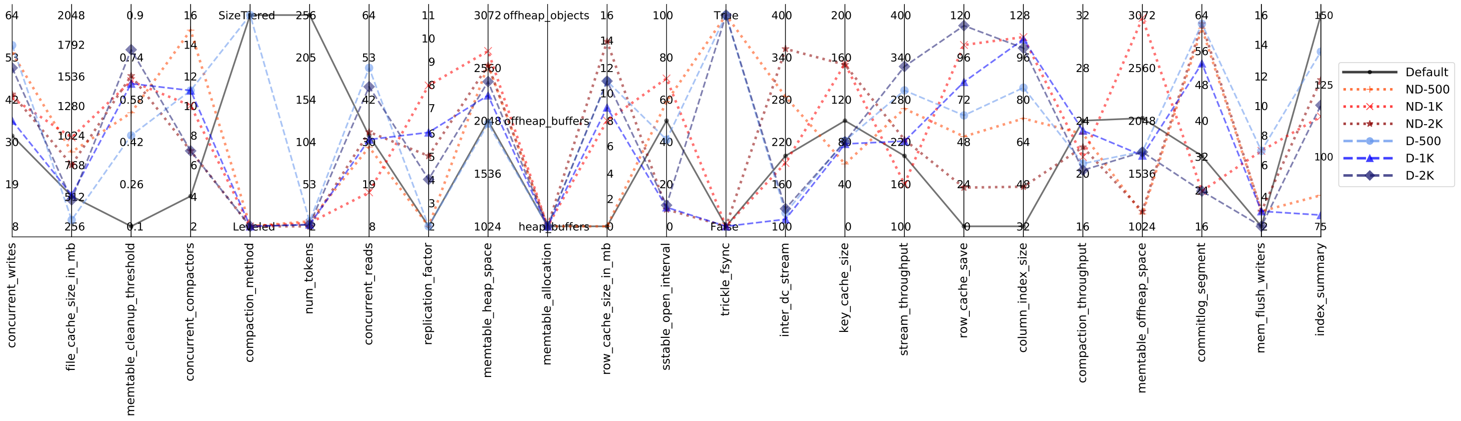

Figure 40: Parallel coordinates plot obtained tuning 23 parameters for workload E. The reported results represent the final configuration obtained after the tuning process. The plot reports the configuration obtained by, from top to bottom, the default configuration, irace without the default configuration (budgets of 500, 1000 and 2000 experiments), and irace with the default configuration (budgets of 500, 1000 and 2000 experiments).

18 Comparison with another methodology

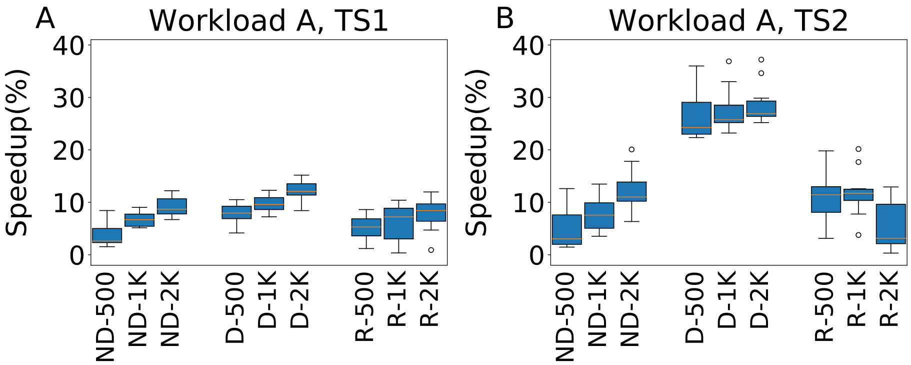

Figure 41: **Comparison of speedups obtained by irace and Rafiki a state-of-the-art methodology with respect to the default configuration tuning 5 parameters for workload A in test scenario 1 and test scenario 2.** The results show the measurement by testing the final configuration over workload A, repeating each experiment ten times. The boxplots report the speedup obtained by, from left to right, irace without the default configuration (budgets of 500, 1000 and 2000 experiments), irace with the default configuration (budgets of 500, 1000 and 2000 experiments), and Rafiki (budgets of 500, 1000 and 2000 experiments).

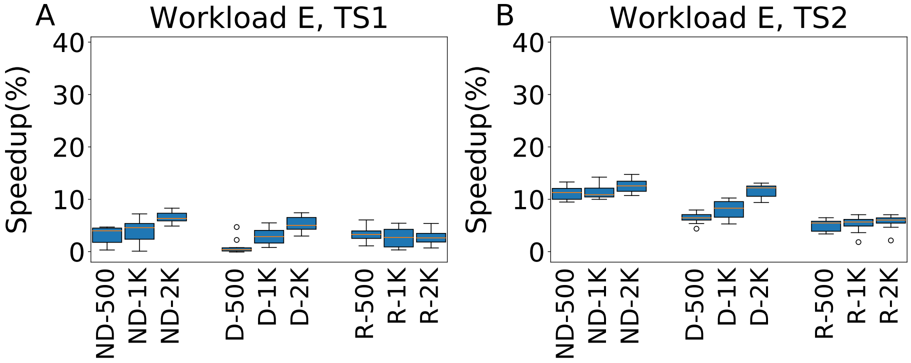

Figure 42: **Comparison of speedups obtained by irace and Rafiki a state-of-the-art methodology with respect to the default configuration tuning 5 parameters for workload E in test scenario 1 and test scenario 2.** The results show the measurement by testing the final configuration over workload E, repeating each experiment ten times. The boxplots report the speedup obtained by, from left to right, irace without the default configuration (budgets of 500, 1000 and 2000 experiments), irace with the default configuration (budgets of 500, 1000 and 2000 experiments), and Rafiki (budgets of 500, 1000 and 2000 experiments).

19 Statistical analysis

19.1 irace vs default configuration, all six workloads

**Configuration**	**ND500**	**ND1000**	**ND2000**	**D500**	**D1000**	**D2000**
WL6-5P-TS1						
WLA	**0.001953125**	**0.001953125**	**0.001953125**	**0.01367188**	**0.001953125**	**0.001953125**
WLB	0.08398438	**0.04882812**	**0.005859375**	**0.009765625**	**0.01367188**	**0.001953125**
WLC	0.1308594	**0.009765625**	**0.00390625**	**0.001953125**	**0.03710938**	**0.001953125**
WLD	**0.01367188**	**0.01367188**	**0.001953125**	**0.001953125**	**0.001953125**	**0.001953125**
WLE	**0.009765625**	1	**0.001953125**	0.06445312	**0.001953125**	**0.001953125**
WLF	**0.001953125**	**0.001953125**	**0.00390625**	**0.00390625**	**0.02734375**	**0.02734375**
WL6-5P–TS2						
WLA	**0.009765625**	**0.001953125**	**0.001953125**	**0.001953125**	**0.001953125**	**0.001953125**
WLB	**0.00390625**	**0.001953125**	**0.001953125**	**0.001953125**	**0.001953125**	**0.001953125**
WLC	**0.001953125**	**0.001953125**	**0.001953125**	**0.001953125**	**0.001953125**	**0.001953125**
WLD	**0.001953125**	**0.00390625**	**0.001953125**	**0.00390625**	**0.001953125**	**0.001953125**
WLE	**0.04882812**	**0.001953125**	**0.001953125**	**0.001953125**	**0.001953125**	**0.001953125**
WLF	0.08398438	**0.001953125**	**0.001953125**	**0.001953125**	**0.001953125**	**0.001953125**
WL6-23P–TS1						
WLA	**0.001953125**	**0.001953125**	**0.001953125**	**0.001953125**	**0.001953125**	**0.001953125**
WLB	**0.001953125**	**0.001953125**	**0.001953125**	**0.001953125**	**0.001953125**	**0.001953125**
WLC	**0.00390625**	**0.001953125**	**0.001953125**	**0.001953125**	**0.001953125**	**0.001953125**
WLD	**0.001953125**	**0.001953125**	**0.001953125**	**0.001953125**	**0.001953125**	**0.001953125**
WLE	**0.04882812**	1	**0.001953125**	0.6953125	0.1308594	**0.001953125**
WLF	**0.001953125**	**0.001953125**	**0.001953125**	**0.001953125**	**0.001953125**	**0.001953125**
WL6-23P–TS2						
WLA	**0.001953125**	**0.001953125**	**0.001953125**	**0.001953125**	**0.001953125**	**0.001953125**
WLB	**0.001953125**	**0.001953125**	**0.001953125**	**0.001953125**	**0.001953125**	**0.001953125**
WLC	0.1601562	**0.005859375**	**0.001953125**	**0.009765625**	**0.001953125**	**0.001953125**
WLD	**0.001953125**	**0.001953125**	**0.001953125**	**0.001953125**	**0.001953125**	**0.001953125**
WLE	0.8457031	0.1054688	**0.001953125**	0.375	**0.01367188**	**0.001953125**
WLF	**0.00390625**	**0.001953125**	**0.001953125**	**0.001953125**	**0.001953125**	**0.001953125**

Table 2: Pairwise Wilcoxon test comparing the results in terms of throughput obtained using irace with those obtained using the default Cassandra configuration across the six workloads of YCSB (A to F). The table is divided for the number of parameters considered (5 or 23), and the test scenario in which the experiments were run (TS1 or TS2). We report the statistical test results for budgets of 500, 1000 and 2000 experiments per tuning including (D-x) and not including (ND-x) the default configuration as an initial configuration for irace. Results in boldface indicate statistically significant results for a p-value threshold of 0.05.

19.2 irace vs default configuration, selected workloads

Configuration	ND500	ND1000	ND2000	D500	D1000	D2000
WLA–5P–TS1	**0.001953125**	**0.001953125**	**0.001953125**	**0.001953125**	**0.001953125**	**0.001953125**
WLA–5P–TS2	**0.001953125**	**0.001953125**	**0.001953125**	**0.001953125**	**0.001953125**	**0.001953125**
WLA–23P-TS1	1	**0.009765625**	**0.001953125**	**0.001953125**	**0.001953125**	**0.001953125**
WLA–23P-TS2	**0.001953125**	**0.001953125**	**0.001953125**	**0.001953125**	**0.001953125**	**0.001953125**
WLE–5P–TS1	**0.001953125**	**0.001953125**	**0.001953125**	**0.00390625**	**0.001953125**	**0.001953125**
WLE–5P–TS2	**0.001953125**	**0.001953125**	**0.001953125**	**0.001953125**	**0.001953125**	**0.001953125**
WLE–23P-TS1	**0.00390625**	**0.001953125**	**0.001953125**	**0.001953125**	**0.001953125**	**0.001953125**
WLE–23P–TS2	**0.001953125**	**0.001953125**	**0.001953125**	**0.001953125**	**0.001953125**	**0.001953125**

Table 3: Pairwise Wilcoxon test comparing the results in terms of throughput obtained using irace with those obtained using the default Cassandra configuration. The first column of the table shows the workload (A or E from YCSB), the number of parameters considered (5 or 23), and the test scenario in which the experiments were run (TS1 or TS2). The remaining columns show the statistical test results for budgets of 500, 1000 and 2000 experiments per tuning including (D-x) and not including (ND-x) the default configuration as an initial configuration for irace. Results in boldface indicate statistically significant results for a p-value threshold of 0.05.

19.3 irace vs Rafiki

Configuration	ND-500	ND-1000	ND-2000	D-500	D-1000	D-2000
WLA–TS1	0.193359375	0.556640625	0.431640625	**0.005859375**	**0.001953125**	**0.00390625**
WLA–TS2	**0.001953125**	**0.00390625**	**0.00390625**	**0.001953125**	**0.001953125**	**0.001953125**
WLE–TS1	0.845703125	0.556640625	**0.001953125**	**0.001953125**	0.76953125	**0.005859375**
WLE–TS2	**0.001953125**	**0.001953125**	**0.001953125**	**0.013671875**	**0.001953125**	**0.001953125**

Table 4: Pairwise Wilcoxon test comparing the results in terms of throughput obtained using irace with those obtained using Rafiki, for 5 parameters. The first column of the table shows the workload (A or E from YCSB) and the test scenario in which the experiments were run (TS1 or TS2). The remaining columns show the statistical test results for budgets of 500, 1000 and 2000 experiments per tuning including (D-x) and not including (ND-x) the default configuration as an initial configuration for irace. Results in boldface indicate statistically significant results for a p-value threshold of 0.05.

20 Time of each irace experiment.

**Setup**	**W6, 5**	**W6, 23**	**WA, 5**	**WA, 23**	**WE, 5**	**WE, 23**
ND-500	8h45m	8h34m	8h54m	9h26m	10h25m	10h54m
ND-1K	18h45m	19h23m	17h50m	19h6m	21h58m	24h1m
ND-2K	41h14m	38h19m	35h57m	37h20m	44h1m	47h28m
D-500	9h21m	9h38m	8h57m	8h12m	10h40m	12h56m
D-1K	19h29m	19h20m	17h20m	17h37m	21h44m	24h5m
D-2K	37h36m	37h49m	36h3m	37h6m	44h14m	48h1m

Table 5: **Time of each irace experiment.**

References

[1] Ashraf Mahgoub, Paul Wood, Sachandhan Ganesh, Subrata Mitra, Wolfgang Gerlach, Travis Harrison, Folker Meyer, Ananth Grama, Saurabh Bagchi, and Somali Chaterji. Rafiki: a middleware for parameter tuning of nosql datastores for dynamic metagenomics workloads. In *Proceedings of the 18th ACM/IFIP/USENIX Middleware Conference*, pages 28–40, 2017.
